# Fault-class coverage–aligned combined training for AFDD of AHUs across multiple buildings

**DOI:** 10.1038/s41598-025-24959-9

**Published:** 2025-11-21

**Authors:** Seunghyeon Wang

**Affiliations:** Institute for Environmental Design and Engineering, University College London, London, WC1H 0NN UK

**Keywords:** Air handling units, Automated fault diagnosis detection, Class-coverage alignment, Deep learning, Attention-based methods, Engineering, Health care, Mathematics and computing

## Abstract

Deep learning–based Automated Fault Detection and Diagnosis (AFDD) for Air Handling Units (AHUs) has often performed well. However, most prior studies relied on single-building datasets and fixed feature schemas, limiting applicability to new sites. This study evaluated TabTransformer and TabNet on a unified multi-building dataset pooling auditorium, hospital, and office data to examine coverage-aligned combined training across buildings. Extensive hyperparameter optimization covering 3240 models and systematic analyses were performed, including checks for underfitting and overfitting, assessment of validation–test variation, best-model selection, attention heatmaps, and comparisons with non-attention baselines. The optimized TabNet trained on unified data achieved, for the auditorium, an F1 score of 97.43% and an accuracy of 97.91%; for the hospital, an F1 score of 92.01% and an accuracy of 92.50%; and for the office, an F1 score of 92.25% and an accuracy of 92.46%. Single-building TabNet baselines reached 96.82% F1 and 97.37% accuracy for the auditorium, 95.40% F1 and 97.21% accuracy for the hospital, and 96.27% F1 and 97.29% accuracy for the office. Across all three buildings, gains from combined training arose primarily under strong coverage alignment of fault classes between the training and target sets; when alignment was weak, the gains diminished.

**Supplementary Information:**

The online version contains supplementary material available at 10.1038/s41598-025-24959-9.

## Introduction

Air Handling Units (AHUs) in Heating, Ventilation, and Air Conditioning (HVAC) systems play a key role in regulating and distributing conditioned air to sustain desirable indoor environments, including appropriate temperature, humidity, ventilation, and air quality levels^1–3^. Automated Fault Detection and Diagnosis (AFDD) for AHUs involves the ongoing monitoring of system components—such as sensors, actuators, and controllers—to rapidly identify abnormal operating conditions or equipment faults^4,5^.

Data-driven AFDD approaches, particularly those based on supervised machine learning, depend heavily on the availability of labeled datasets^6^. Lab- or simulation-based data are easier to obtain but miss real operational complexity; real building data capture authentic fault patterns yet are costly to collect and label, requiring substantial domain expertise^7–9^. However, many existing AFDD models suffer from poor transferability across buildings, with performance dropping sharply when models trained on one site are applied to others. This is largely due to the limited diversity of single-source datasets, compounded by variations in operational schedules, environmental conditions, and sensor configurations^10–12^.

Expanding training to unified datasets that integrate information from multiple buildings can help address this issue. Such datasets expose models to a wider variety of operating conditions and fault scenarios, enabling them to develop more generalized, building-independent fault representations that improve performance in unfamiliar environments^13^. However, unified datasets bring their own challenge: sensor configurations frequently vary in both type and number across buildings. Conventional supervised models, which typically require fixed-length inputs, are not well suited to handle this variability.

Attention-based neural networks offer a promising solution, as they are inherently capable of processing variable-length inputs. This allows them to flexibly adapt to differing sensor setups without the need for extensive manual preprocessing or feature alignment^14^. As a result, attention mechanisms were a strong fit for unified training across buildings with heterogeneous sensors. To examine the role of coverage alignment of classes in multi-building AFDD, the study evaluated attention-based architectures trained and tested on real operational datasets from three building types—an auditorium, a hospital, and an office. Coverage alignment of classes was defined as the overlap between the class distributions of the training sources and the target building.

The main contributions of this paper are summarized as follows:

1. Real operational datasets were collected over one year from 13, 8, and 20 AHUs installed in an auditorium, hospital, and office building, respectively, each with distinct sensor configurations.
2. Operating-condition labels were defined and annotated—seven in the auditorium and four in both the hospital and office—each set comprising one normal state and multiple fault states.
3. Attention-based models (TabTransformer, TabNet) were optimized via systematic hyperparameter sweeps, and 3240 configurations were evaluated to select the best models.
4. Analyses included checks for underfitting and overfitting, assessment of between-method variability, best-model selection, attention heatmaps, and comparative evaluation against 2592 single-building baseline models.
5. Findings showed that performance under unified training depended on coverage alignment of classes: accuracy was high when target-building fault coverage matched the training set and declined under mismatched coverage.

## Literature review

### Supervised learning using real operational data

Previous supervised learning studies can be divided into three: simulation programs, laboratory experiments, or real operation data. Simulation data generated using non-proprietary, physical model-based software have been widely employed in HVAC research^15–19^. Although simulation-based datasets provide convenient and controlled environments for developing and validating FDD models, these models often exhibit reduced performance in real-world settings due to inherent discrepancies between simulated and actual operational conditions^6^. Controlled simulations generally fail to fully represent real-world complexities and variability, limiting the effectiveness of models when deployed in practice.

Laboratory datasets are collected from controlled experimental setups that mimic actual HVAC operation^20–24^ While laboratory data offer greater realism compared to purely simulated datasets, controlled laboratory settings still cannot fully capture the variability and unpredictability present in real operational environments^12^. Factors such as weather fluctuations and occupant behavior, which significantly influence HVAC performance, are typically absent or controlled in laboratory conditions, thereby limiting the real-world generalizability of laboratory-based models^6^.

Real operational datasets, on the other hand, provide higher realism as they capture the actual complexities and operational variability present in HVAC systems^6^. Nonetheless, generating precise labels for these datasets is challenging due to subtle indications of faults, the infrequent nature of specific fault conditions causing class imbalance, and the considerable domain expertise and effort necessary for accurate data annotation.

### Research trends in supervised learning

Although real operational datasets provide notable advantages, previous AFDD research has primarily relied upon simulated or laboratory-generated data^25–27^. To overcome labeling difficulties inherent to real-world data, researchers have pursued alternative methods, such as semi-supervised learning, which significantly reduces the required amount of labeled data, and fully unsupervised learning approaches that operate without any labeled examples.

Key semi-supervised techniques include AutoEncoders (AEs)^28,29^ and Generative Adversarial Networks (GANs)^30–32^. Additionally, unsupervised approaches involving Principal Component Analysis (PCA)^17,33,34^ and transformer encoder architectures^35^ have been explored, alongside specialized noise reduction methods employing GANs^36^, PCA^37^, and AEs^38,39^.

However, the validation of these alternative methods has typically been limited to datasets that fail to represent the intricate complexities and realistic scenarios of operational HVAC systems. This limitation restricts their practical utility. Consequently, there is a pressing requirement to develop robust supervised learning techniques explicitly trained and validated on real-world operational datasets sourced directly from active HVAC systems. Such methods could establish reliable benchmarks, facilitating accurate evaluations and improvements of models derived from simulations or laboratory experiments.

Most prior AFDD studies rely on operational data from individual buildings, limiting transfer across sites. Recent work has begun to cover diverse facilities—e.g., data centers^40^, hospitals^11^, auditoriums^13^, and university campuses^41^. A unified, multi-building dataset enhances generalization by exposing models to diverse operating regimes, sensor schemas, and fault manifestations, thereby reducing site-specific overfitting and improving robustness on unseen buildings. By broadening the data distribution (seasonal loads, schedules, climates, maintenance practices) and spanning varied sensor types, counts, and calibrations, it drives the model to learn building-agnostic fault signatures rather than site-specific artifacts.

This study contributes such a unified, real-operation dataset and shows that unified training yields high accuracy—establishing a practical path toward building-agnostic AFDD. To preserve these gains, unified training should be paired with schema-tolerant encoders (e.g., attention/set/graph with sensor-type embeddings and masking) and coverage-aware evaluation to manage remaining mismatches in sensors and fault labels.

### Attention-based method

Attention-based neural networks have gained popularity due to their flexibility and efficiency in processing variable-length input sequences. Attention mechanisms enable models to dynamically identify and emphasize crucial portions or features within input data, which is especially advantageous for modeling complex temporal interactions and long-term dependencies inherent in time-series data^42,43^. Unlike traditional neural networks, attention models adaptively adjust the significance assigned to individual data points, significantly enhancing predictive accuracy and interpretability by highlighting data features indicative of specific faults^44^.

In AFDD applications, attention mechanisms are particularly beneficial because they enable detection of subtle anomalies and operational variations in AHUs even in noisy, real-world environments. The inherent flexibility of attention-based models makes them suitable for robust fault detection across diverse settings, including hospitals, auditoriums, and large-scale office buildings, where prompt and accurate fault diagnosis is essential for operational reliability and efficiency.

To the best of the authors’ knowledge, this is the first study to train attention-based AFDD models exclusively on operational AHU data collected across multiple environments—an auditorium, a hospital, and a large office building. By consolidating these datasets, the study demonstrates the effectiveness of attention mechanisms for handling variable-length sensor inputs and examines how performance depends on the coverage alignment of fault classes between training and target buildings; in practice, gains are largest under strong alignment.

## Proposed methodology

**Fig. 1:**
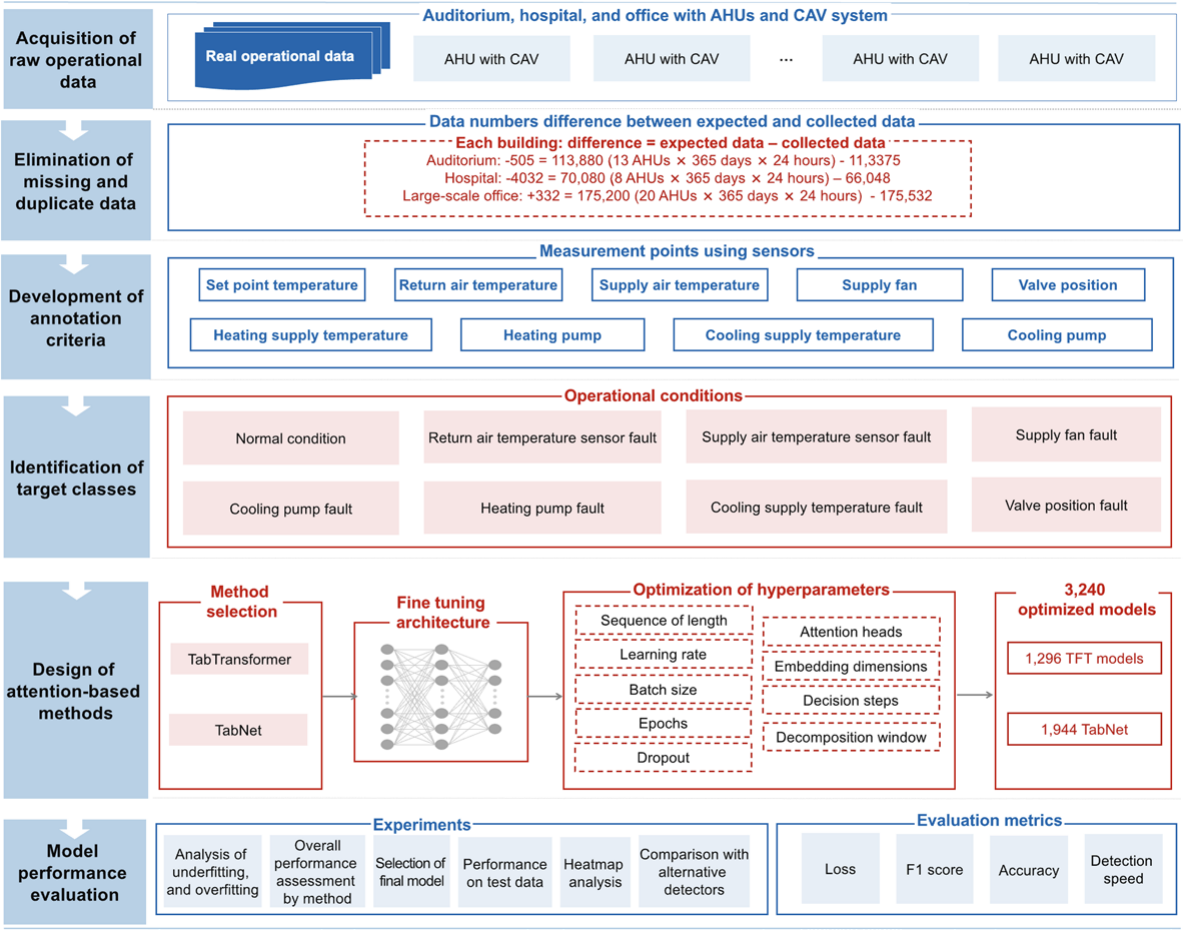
Workflow of proposed methodology.

As depicted in Fig. 1, the attention-based approaches proposed in this research is systematically structured into six sequential steps, starting from acquisition raw operational data and culminating in model performance evaluation. The following subsections detail the selected discretization strategies for fault classification, outline the comparative methods adopted, and provide justifications for the chosen evaluation criteria.

### Acquisition of raw operational data

Each building category employed multiple AHUs, each differing in quantity, specifications, and sensor arrangements specifically designed for effective AFDD across distinct rooms or spaces. Originally, sensor readings were captured at one-minute intervals. However, to facilitate practical analysis at an hourly scale, these minute-level readings were consolidated into hourly averages. This approach significantly streamlined data storage and alleviated the management complexities commonly encountered with extensive, high-frequency datasets in operational scenarios. Each sensor type was calibrated as outlined below. Table 1 provides sensor descriptions, units, and calibration ranges with accuracy. While the sensor types remained uniform across different buildings, the exact number of sensors varied slightly due to each building’s distinct characteristics. Further dataset specifics for each building type are presented below.

**Table 1:** Description of measurement points for AHU datasets.

Sensor or aggregated metric	Description	Unit	Auditorium (15 points)	Hospital (9 points)	Office (18 points)	Sensor accuracy
Installed sensors						
Set point temperature	Temperature setting specified by the operator	°C	✓	✓	✓	± 0.2 °C
Return air temperature	Air temperature returning from ventilated areas	°C	✓	✓	✓	± 0.2 °C
Supply air temperature	Air temperature delivered by the AHU	°C	✓	✓	✓	± 0.2 °C
Supply fan	Operational condition of the AHU supply fan	%	✓	✓	✓	± 1%
Valve position	Valve position condition within the AHU system	%	✓	✓	✓	± 2%
Heating supply temperature 1	Temperature in heating system’s supply line 1	°C	✓	✓	✓	± 0.2 °C
Heating supply temperature 2	Temperature in heating system’s supply line 2	°C			✓	
Heating pump 1	Operational condition of heating pump 1	%	✓	✓	✓	± 1%
Heating pump 2	Operational condition of heating pump 2	%	✓		✓	
Heating pump 3	Operational condition of heating pump 3	%	✓		✓	
Cooling supply temperature 1	Cooling supply temperature sensor 1	°C	✓	✓	✓	± 0.2 °C
Cooling supply temperature 2	Cooling supply temperature sensor 2	°C	✓		✓	
Cooling pump 1	Operational condition of cooling pump 1	%	✓	✓	✓	± 1%
Cooling pump 2	Operational condition of cooling pump 2	%	✓		✓	
Cooling pump 3	Operational condition of cooling pump 3	%			✓	
Cooling pump 4	Operational condition of cooling pump 4	%			✓	
Aggregated Metrics						
Total heating pump	Aggregated operational condition of heating pumps	%	✓		✓	
Total cooling pump	Aggregated operational condition of cooling pumps	%	✓		✓

- Auditorium

Data collection took place at the Sejong Arts Center (located at 21 Gungnipbangmulgwan-ro, Sejong-si), which encompasses an area of 16,186 m² spanning from basement level 1 (B1) up to the sixth floor (6 F). The data were systematically gathered over the full calendar year of 2022, from January 1 through December 31. The center houses 13 AHUs, each equipped with specialized cooling and heating functionalities tailored to distinct building zones, as presented in Table S.1. Each AHU was integrated with a network of 15 different sensors. These AHUs continuously collected data at hourly intervals throughout the year, culminating in an extensive dataset totaling 113,880 hourly data points.

- Hospital

Data were collected from the New Wing of the National Cancer Center Hospital (323 Ilsan-ro, Ilsandong-gu, Goyang-si, Gyeonggi-do), which has a total area of 18,900 m², ranging from the second basement level up to the fifth floor. The facility, completed in October 2020, underwent continuous data collection throughout the year 2022, specifically from January 1 to December 31. The building features eight AHUs, each fitted with dedicated cooling and heating coils, as described in Table S.2. Nine distinct sensor variables were integrated into the AHU system. The data collected hourly over the entire year generated an extensive dataset consisting of 70,080 hourly observations.

- Office

Data collection was carried out in a large office building situated at 48 Gwacheon-daero 7na-gil, Gwacheon-si, Gyeonggi-do. The facility spans 50,966 m², extending from basement level 4 up to the sixth floor. Continuous data collection occurred from December 1, 2023, through November 30, 2024. The building houses 20 AHUs, each equipped with dedicated heating and cooling coils, further detailed in Table S.3. Eighteen distinct sensor variables were integrated. The hourly dataset collected over this period contains 175,532 entries, slightly surpassing the expected theoretical total of 175,200 hourly observations (computed as 20 AHUs × 365 days × 24 h). This discrepancy likely results from supplementary logging or operational monitoring activities that occurred during the data collection period.

### Elimination of missing and duplicate data

Initially, data collection involved hourly measurements from multiple AHUs across three distinct buildings over an entire year (365 days). The expected theoretical data points were computed by considering the number of AHUs, the days in a year, and the hourly recording frequency. However, upon comparing actual collected datasets with these theoretical counts, some discrepancies emerged, as illustrated in Table 2.

Specifically, the datasets from the auditorium and hospital contained fewer records than expected, suggesting the presence of missing values across certain independent variables. The auditorium data lacked 505 hourly records, whereas the hospital data was missing 4032 entries. These missing records likely stemmed from various issues such as sensor malfunctions, temporary interruptions in data logging, or communication failures during the data collection process^45^.

In contrast, the office building dataset had 332 more records than theoretically predicted. Further analysis revealed these surplus entries were duplicate measurements resulting from logging errors or synchronization issues. After eliminating these duplicates, the office dataset aligned precisely with the theoretically anticipated data count.

**Table 2:** Comparison between expected and collected data points, and identified data quality issues.

Dataset	Expected data points	Collected data points	Difference	Issue type
Auditorium	113,880 (13 AHUs × 365 days × 24 h)	11,3375	– 505	Missing entries
Hospital	70,080 (8 AHUs × 365 days × 24 h)	66,048	– 4032	Missing entries
Office	175,200 (20 AHUs × 365 days × 24 h)	175,532	332	Duplicated entries

In this study, both missing and duplicate records were removed. Although imputation is an option when data are sparse, exclusion was chosen because the dataset was sufficiently large to support robust analysis without imputation. This choice is consistent with prior studies^11,13^, which report strong performance when training datasets are sufficiently large.

### Development of annotation criteria

Operational categories and thresholds. We defined seven operational categories for annotation—one normal condition and six fault conditions: supply fan fault, cooling pump fault, heating pump fault, return-air temperature sensor fault, supply-air temperature sensor fault, and valve-position fault. Although general guidance exists to distinguish normal from faulty behavior, not all faults have well-established numerical thresholds. As noted by Wang et al.^12^, sensor-related faults such as RATSF and SATSF often have broadly accepted numeric criteria, whereas actuator-related faults like VPF are typically identified using more qualitative rules rather than precise ranges.

In practice, numeric criteria must be tailored to each AHU to reflect differences in configuration, manufacturer specifications, and operating environment. In this study, six HVAC specialists (each with >20 years of experience) derived building-appropriate thresholds following ASHRAE-recommended practices, calibrated for typical AHUs. Table S4 reports the condition-specific criteria and thresholds, which follow and refine^12^. Table S5 illustrates the labeling procedure with an auditorium example.

### Indentification of target classes

Four annotators possessing extensive expertise exceeding 20 years in mechanical engineering and HVAC AFDD manually annotated the dataset using Excel. Table S6 presents the distribution of fault conditions within each dataset, emphasizing variations resulting from the differing frequencies of specific faults. Interestingly, despite their inclusion in the annotation criteria, certain faults were not detected in particular buildings:

- Cooling pump and heating pump faults were not observed in the hospital and office buildings.
- Cooling supply temperature fault was identified only once within the hospital building.

**Fig. 2:**
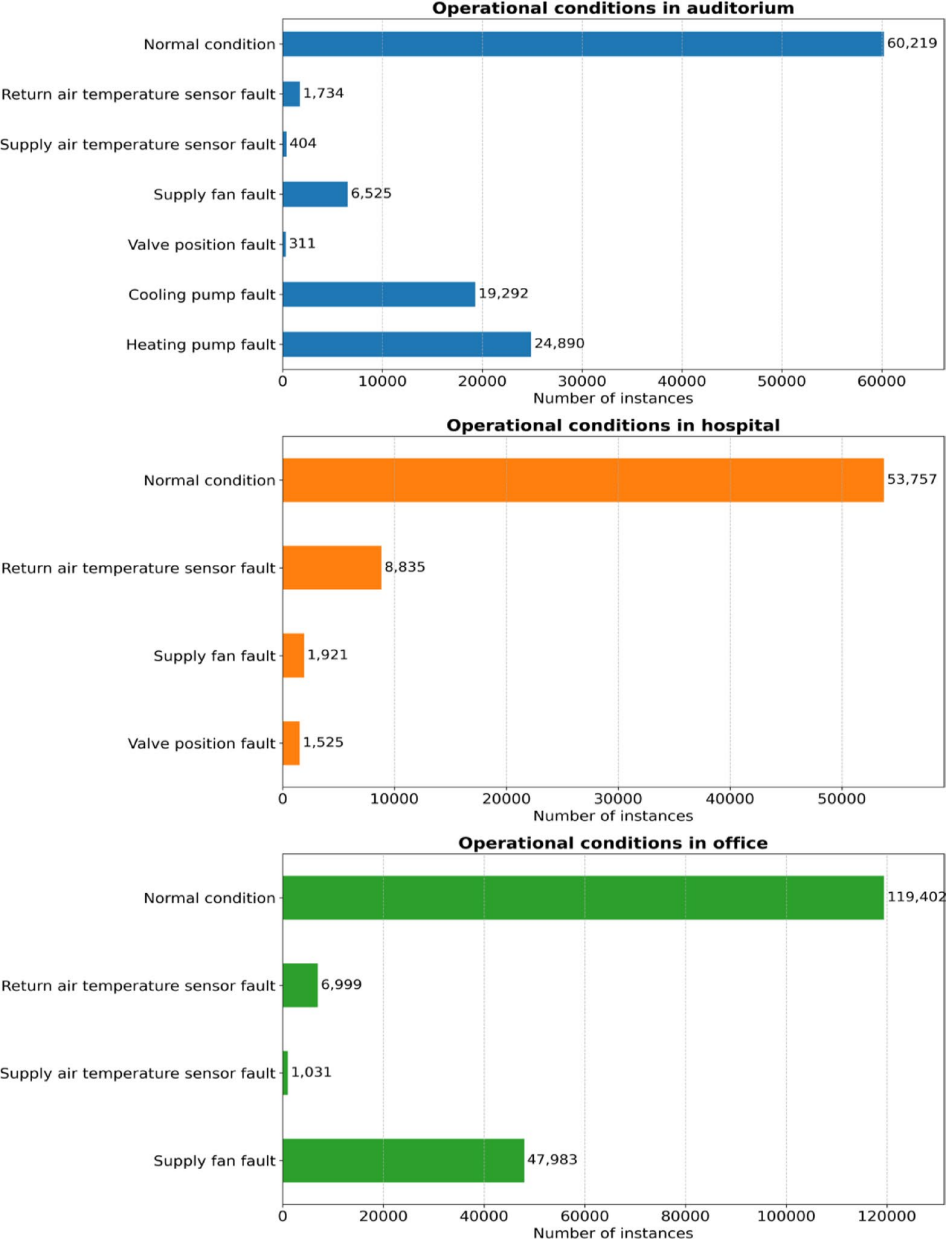
Distribution of target classes with numbers in each building type.

Certain fault conditions either were not observed or appeared very rarely during the data collection period, complicating the creation of robust datasets for model training, validation, and testing. As a result, operational classes with fewer than 300 occurrences were omitted from the analysis. Figure 2 provides a visual representation of the data distribution across the target operational conditions.

### Design of attention-based methods

The inherent variability across datasets and the differing strengths of various algorithms highlight the importance of exploring and comparing multiple tabular-based methods^46,47^. Such comparative assessments facilitate the selection of optimal models that provide robust performance and enhanced ability to generalize.

In this study, two sophisticated tabular-focused methods specifically tailored for structured datasets are investigated: TabTransformer^48^ and TabNet^49^. These methods were chosen due to their demonstrated efficacy and robustness across complex and dynamic applications, including demand prediction, anomaly detection, and fault diagnosis^50^. Despite both approaches incorporating attention mechanisms, they significantly differ regarding their internal attention designs, techniques for feature decomposition, and capabilities for effectively processing lengthy data sequences. Detailed descriptions and critical comparisons of these transformer-based techniques are elaborated upon in the following sections, with their principal distinctions succinctly summarized in Table 3.

**Table 3:** Summary of distinct characteristics in tabular-based transformer model.

Aspect	TabTransformer	TabNet
Attention mechanism	Full self-attention	Sparse feature attention
Sequence handling efficiency	Moderate	High
Feature selection capability	No	Yes
Computational demand	Moderate	Lower
Time series handling capability	Moderate	Strong

#### TabTransformer

**Fig. 3:**
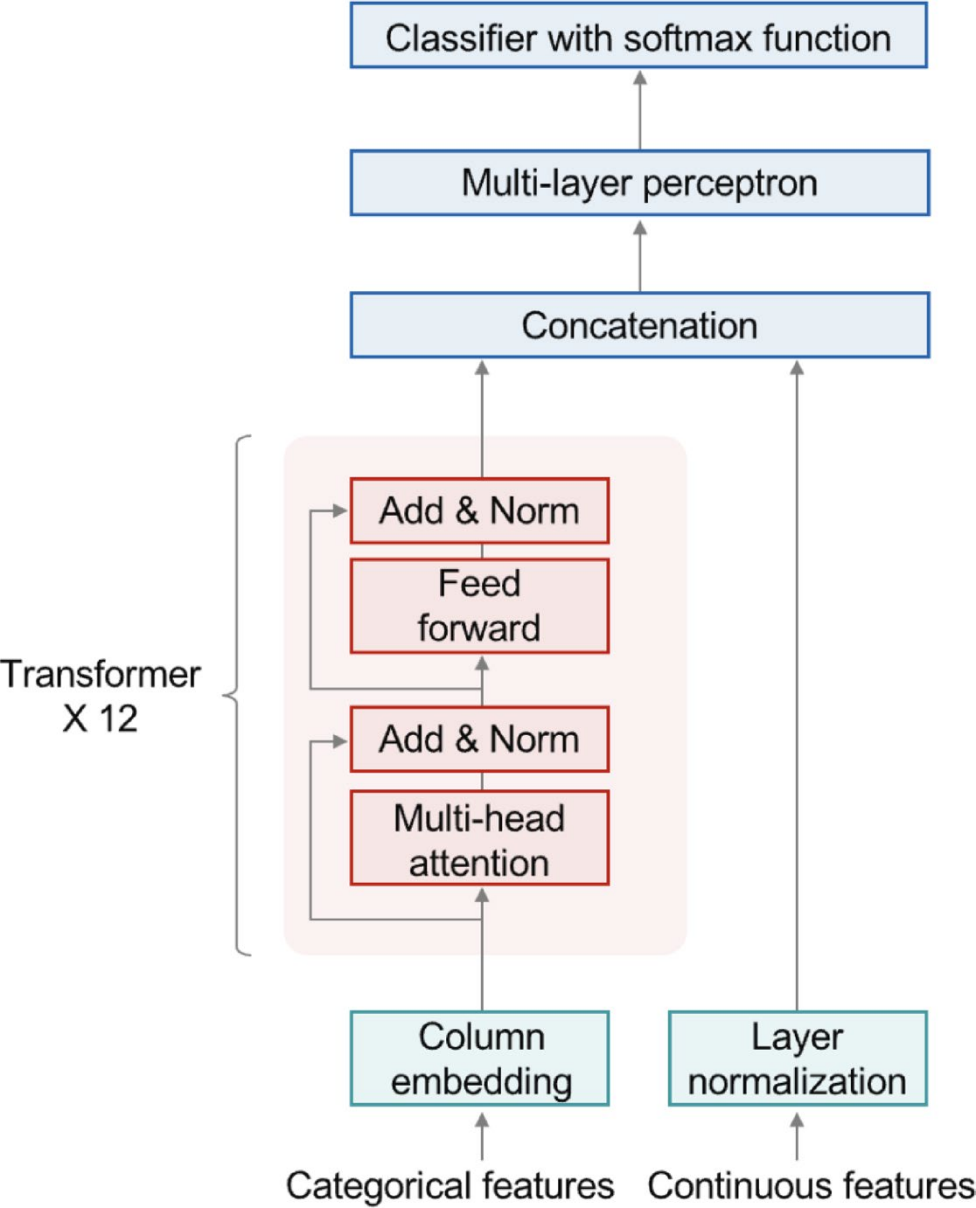
Workflow of TabTransformer.

As depicted in Fig. 3, the TabTransformer is specifically tailored to handle tabular data by embedding categorical variables using transformer-based layers, effectively capturing their interrelationships. It integrates a complete self-attention mechanism, enabling extensive modeling of interactions among categorical features. Continuous variables, in contrast, undergo normalization independently before being merged with categorical embeddings through concatenation^51^. Although its robust attention architecture excels at identifying intricate feature interactions, it leads to moderately increased computational complexity^12,52^. Furthermore, TabTransformer does not explicitly perform feature selection, thereby inferring feature importance implicitly rather than directly. As a result, this method is particularly effective for datasets with rich categorical interactions but exhibits moderate levels of interpretability and computational efficiency.

#### TabNet

**Fig. 4:**
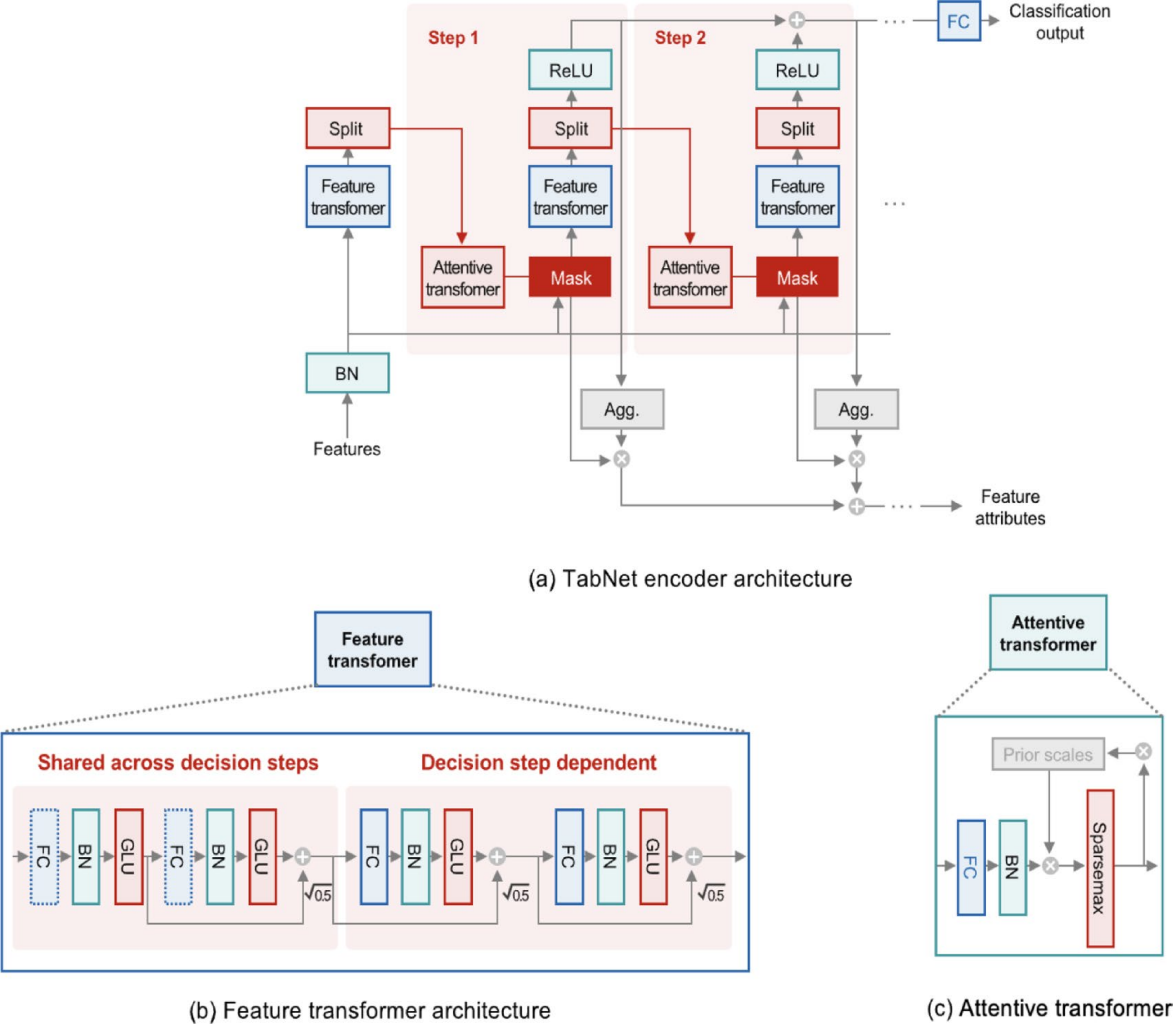
Workflow of TabNet.

As illustrated in Fig. 4, TabNet utilizes a sparse attention mechanism known as Sparsemax, specifically optimized to facilitate explicit feature selection. This attention strategy dynamically emphasizes the most critical features, thereby significantly enhancing interpretability through clear identification of feature importance. Both continuous and categorical variables undergo unified processing via iterative decision steps that incorporate feature and attentive transformers, progressively refining feature relevance and prominence^43,49^. The explicit feature selection capability inherent to TabNet reduces computational load relative to full self-attention models, resulting in greater computational efficiency. Additionally, TabNet’s iterative design effectively captures intricate feature interactions, delivering robust performance alongside high interpretability^53^. Consequently, TabNet is highly suitable for real-world AFDD applications involving sensor-based datasets.

#### Fine tuning of architectures

For effective implementation of attention-based approaches (TabTransformer and TabNet) in AFDD tasks, embeddings derived from these models are directed into a Fully Connected (FC) classification module. Typically, this module is structured with an initial dense layer consisting of 128 neurons, succeeded by a dropout layer set at a dropout rate of 0.3. The final classification phase involves an additional dense layer equipped with a softmax activation function, which classifies embeddings into the distinct operational categories of AHUs illustrated in Fig. 2—specifically, seven operational categories.

#### Optimization of hyperparameters

Selecting optimal hyperparameters is essential for achieving the highest performance from transformer-based models, yet exhaustive hyperparameter tuning often demands significant computational resources. A pragmatic alternative is to establish hyperparameter ranges guided by existing research findings and empirical insights^54–56^. Consequently, careful consideration was given to setting hyperparameter intervals and selecting specific parameter values, resulting in various model configurations for TabTransformer and TabNet. Table S7 provides a comprehensive summary of these hyperparameters, distinguishing between those common to both methods and those specific to each, along with the total count of model configurations generated.

### Model performance evaluation

#### F1 score

In AFDD classification tasks, F1 score and accuracy are commonly adopted to assess model performance. The F1 score combines precision and recall into a unified metric, effectively balancing the model’s capacity to identify relevant occurrences (recall) and its precision in minimizing false detections. A detailed definition and discussion are provided in paper^57^. High F1 scores reflect the model’s proficiency in accurately detecting faults while reducing both false positives and false negatives, thereby enhancing system reliability.

#### Accuracy

In this study, two forms of accuracy are reported: average accuracy and overall accuracy. Average accuracy (macro accuracy) is calculated as the unweighted mean of per-class accuracies, giving equal importance to each fault category regardless of its prevalence. Overall accuracy (micro accuracy) is computed as the total number of correct predictions divided by the total number of samples, thereby weighting results according to class frequency. The detailed definitions of these metrics are provided in^58^.

This distinction is particularly important in HVAC AFDD applications, where datasets are often imbalanced due to the low occurrence of certain fault types. Relying solely on overall accuracy can obscure poor performance in rare but operationally critical faults, potentially leading to undetected failures. In contrast, average accuracy ensures that all fault categories, including infrequent yet high-impact faults, are equally represented in the performance evaluation, enabling a more balanced and reliable assessment of diagnostic capability.

#### Detection speed

Detection speed refers to the duration required by a model to evaluate an individual data instance. In this study, detection speed is quantified as the count of data instances analyzed per second, serving as an indicator of computational efficiency across various models in processing sensor data for determining AHU operational states.

## Experimental design

### Dataset Preparation

Upon completing dataset annotation, the collected data were randomly divided into training (60%), validation (20%), and test (20%) subsets. This division ensures an objective and accurate evaluation of the model’s performance using unseen data. However, traditional random splitting methods can unintentionally lead to inadequate representation of minority classes, resulting in biased performance assessments^59,60^. To address this concern, stratified sampling was employed to preserve proportional representation across all classes within each subset.

As previously illustrated in Fig. 2, certain fault conditions either did not occur or occurred infrequently during data collection, leading to varying target classes across different building types. Consequently, some fault categories were excluded for particular buildings due to insufficient data instances, resulting in differences in target classes among buildings. For simplicity and readability, the following abbreviations are utilized throughout this study:

- Normal condition: “Normal”.
- Return Air Temperature Sensor Fault: “RATSF”.
- Supply Air Temperature Sensor Fault: “SATSF”.
- Supply Fan Fault: “SFF”.
- Valve Position Fault: “VPF”.
- Cooling Pump Fault: “CPF”.
- Heating Pump Fault: “HPF”.

By applying stratified sampling, class distributions remain consistent across training, validation, and test subsets. This approach ensures balanced representation, particularly for less frequently occurring classes, reducing potential biases, facilitating more stable model training, and enhancing the reliability and accuracy of subsequent evaluations. The detailed breakdown of class distributions for each subset is provided in Table 4.

**Table 4:** Distribution of training, validation, and test datasets by Building type and fault condition.

Building type	Purpose	Class							
		Normal	RATSF	SATSF	SFF	VPF	CPF	HPF	Total
Auditorium	Training	36,131	1040	242	3915	187	11,576	14,934	68,025
	Validation	12,044	347	81	1305	62	3858	4978	22,675
	Test	12,044	347	81	1305	62	3858	4978	22,675
	Total	60,219	1734	404	6525	311	19,292	24,890	113,375
Hospital	Training	32,253	5301		1153	915			39,622
	Validation	10,752	1767		384	305			13,208
	Test	10,752	1767		384	305			13,208
	Total	53,757	8835		1921	1525			66,038
Office	Training	71,442	4199	619	28,789				105,049
	Validation	23,814	1400	206	9597				35,017
	Test	23,814	1400	206	9597				35,017
	Total	119,070	6999	1,031	47,983				175,083

### Experimental environments

All experiments were executed using a computing environment running Windows 10, equipped with an Intel Core i7-7700HQ processor (operating at 2.80 GHz with 8 threads), an NVIDIA GeForce GTX 3080Ti GPU, and 32 GB of memory. Python was employed for all software implementations, with TensorFlow utilized as the main deep learning framework for model development and execution.

## Results and discussions

### Training and validation results

#### Underfitting, and overfitting

**Fig. 5:**
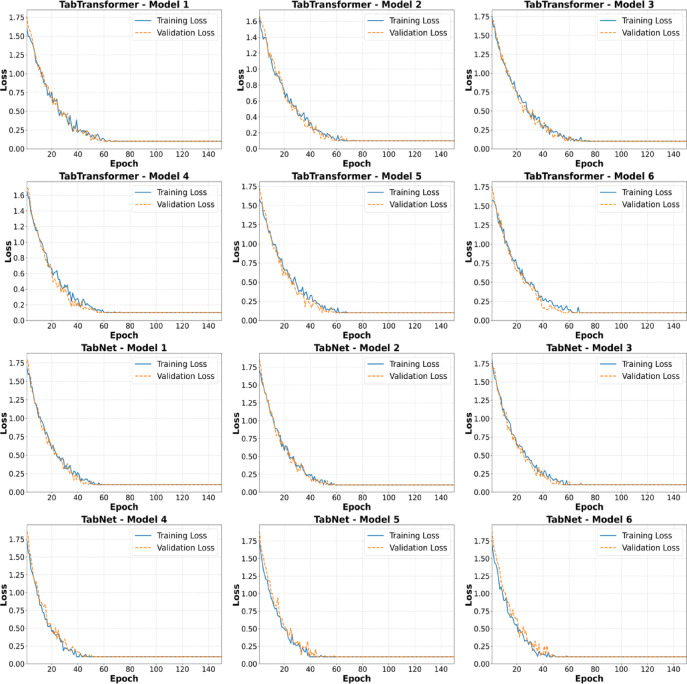
Example of training and validation loss curves for attention-based methods.

In this research, the training and validation loss curves were examined to identify any potential issues related to underfitting or overfitting during model training. Figure 5 demonstrates illustrative cases of these loss curves for each transformer-based model at the maximum number of training epochs, clearly showing their behavior across multiple iterations.

All the models exhibited a steady and consistent reduction in both training and validation losses over the training duration, reflecting stable and effective learning. The persistent downward trend in the training losses indicates that significant underfitting was not present in any model. Moreover, the close similarity and parallel reduction of both training and validation loss curves suggest there was minimal to no overfitting.

#### Analysis of performance variation by methods

##### Analysis of overall performance

**Fig. 6:**
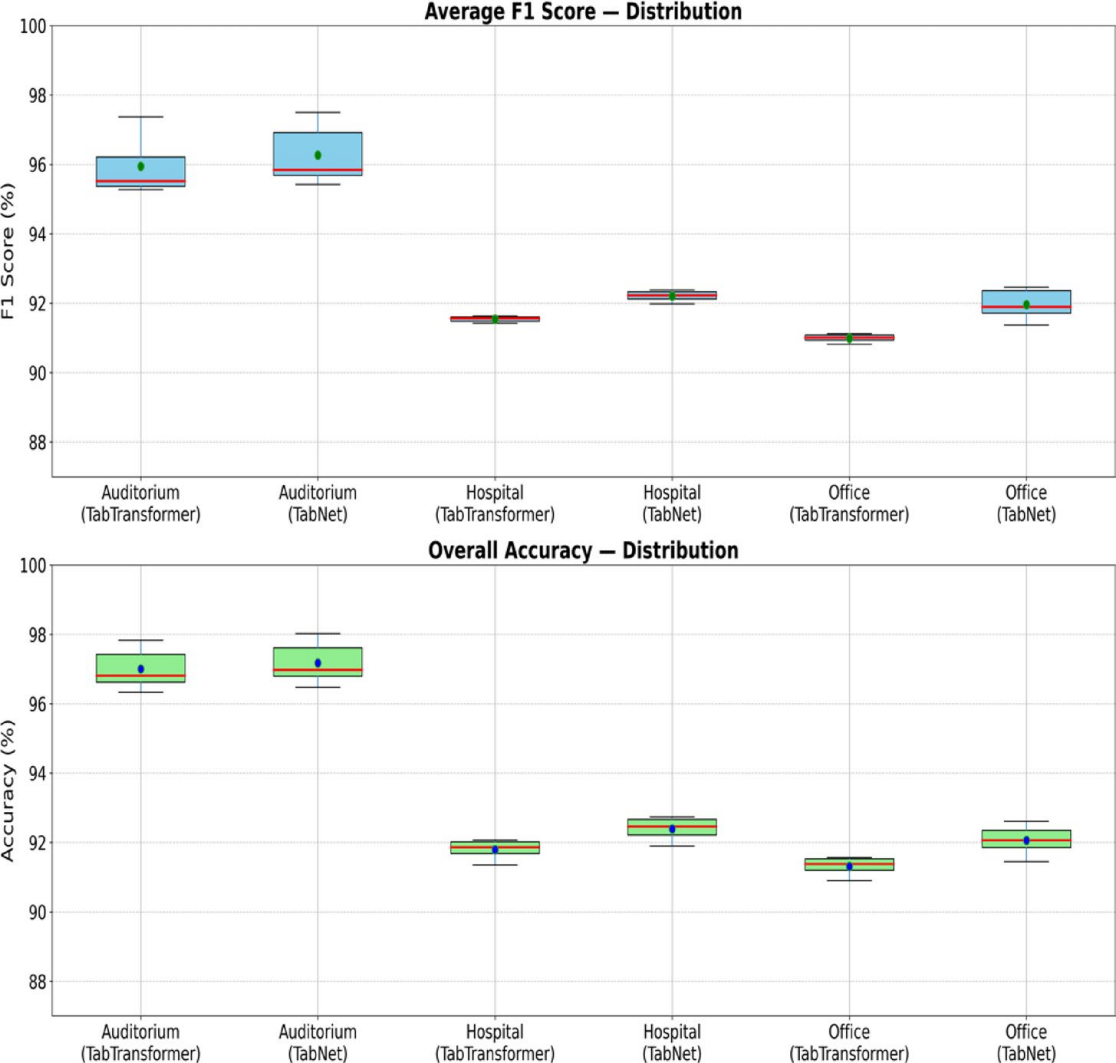
Boxplot distributions of F1 score, and accuracy.

Two attention-based methods—TabTransformer and TabNet—trained on a unified dataset are compared for AFDD across three target buildings (auditorium, hospital, and office); Fig. 6 presents the score distributions, and Table S.8 summarizes the descriptive statistics (macro-F1 over present classes and overall/micro accuracy).

Across all target buildings, TabNet achieved slightly higher mean values than TabTransformer, with gains of + 0.26% F1 and + 0.17% accuracy for the auditorium, + 0.69% F1 score and + 0.62% accuracy for the hospital, and the largest improvements of + 0.96% F1 score and + 0.75% accuracy for the office. Although TabNet consistently outperformed TabTransformer in terms of mean values, the min–max ranges reveal performance overlap between the two models; for example, in the auditorium case, TabTransformer’s maximum accuracy (97.83%) exceeded TabNet’s minimum (96.48%), indicating that under certain hyperparameter configurations TabTransformer can achieve performance comparable to or exceeding TabNet’s lower-bound outcomes. Given that both models were trained on the same unified dataset, these performance variations are attributable not to dataset mismatch but to differences in fault coverage, class distribution, and sensor data characteristics between target buildings.

Standard deviations were small across all cases (< 0.53 for F1, < 0.46 for accuracy), indicating stable performance across hyperparameter settings, with boxplots confirming narrower interquartile ranges in buildings with more comprehensive fault coverage. These findings demonstrate that even when trained on a unified dataset, model performance varies notably across target buildings and is strongly influenced by the breadth and balance of fault coverage, supporting the hypothesis that increasing fault coverage in training data can lead to more consistent generalization across diverse operational contexts.

##### Class-wise performance of best model in each method

Table 5 presents the precision, recall, F1 score, and accuracy at the class level for the best-performing configuration of each method. For the auditorium, both TabTransformer and TabNet achieved high and consistent results across most classes, with precision and recall frequently at or near 100%. The only notable exception was the SFF class, where TabNet’s higher precision (84.97% vs. 83.04%) and F1 score (90.99% vs. 89.87%) indicate an advantage in correctly identifying this class while maintaining low false positives. Class-wise averages for the auditorium were similar (TabNet: 97.38% precision, 97.76% recall, 97.50% F1; TabTransformer: 97.30%, 97.63%, 97.37%), and overall accuracy was marginally higher for TabNet (98.02% vs. 97.83%).

In the hospital, performance gaps were more pronounced, largely due to zero detection in the SATSF, CPF, and HPF classes for both models, reflecting missing or extremely sparse examples in fault coverage. For the remaining classes, TabNet slightly outperformed TabTransformer in most metrics. Notably, for VPF, TabNet’s precision improved from 75.88% to 78.33% and F1 from 83.09% to 84.81%. Overall accuracy was higher for TabNet (92.74% vs. 92.07%), with class-wise average F1 scores also showing improvement (92.38% vs. 91.64%).

In the office, differences between methods were more substantial for certain classes. TabNet achieved higher precision, recall, and F1 scores for Normal, RATSF, SATSF, and SFF, with particularly strong gains in SATSF (F1: 81.72% vs. 78.33%) and SFF (95.85% vs. 95.15%). Similar to the hospital case, some classes (VPF, CPF, HPF) recorded zero recall and F1 scores for both methods due to absent examples in the training dataset for those categories, again pointing to incomplete fault coverage. Class-wise averages in the office were higher for TabNet (92.46% vs. 91.12% F1), and overall accuracy was notably improved (92.61% vs. 91.57%).

Across all three buildings, the largest class-wise differences were driven by how well each method handled classes with moderate-to-low prevalence. TabNet consistently delivered small to moderate gains in these cases, particularly for faults with more complex feature distributions (SFF, SATSF, VPF). By contrast, when a class had zero or near-zero coverage in the training data, neither method generalized: precision was trivially perfect (no false positives) but recall and F1 were zero. These patterns underscore that the completeness of fault-class coverage governs performance even under unified training and indicate that enriching under-represented classes is essential for improving reliability across buildings.

**Table 5:** Precision, recall, F1-score, and accuracy in the class-wise level.

Building	Method	Class	TP	FP	FN	TN	Precision	Recall	F1 score	Accuracy
Auditorium	TabTransformer	Normal	11,783	230	261	10,401	98.09	97.83	97.96	97.83
		RATSF	339	0	8	22,328	100	97.69	98.83	99.96
		SATSF	79	0	2	22,594	100	97.53	98.75	99.99
		SFF	1278	261	27	21,109	83.04	97.93	89.87	98.73
		VPF	60	0	2	22,613	100	96.77	98.36	99.99
		CPF	3775	0	83	18,817	100	97.85	98.91	99.63
		HPF	4870	0	108	17,697	100	97.83	98.9	99.52
		Average					97.3	97.63	97.37	99.38
		Overall								97.83
	TabNet	Normal	11,806	212	238	10,419	98.24	98.02	98.13	98.02
		RATSF	340	0	7	22,328	100	97.98	98.98	99.97
		SATSF	79	1	2	22,593	98.75	97.53	98.14	99.99
		SFF	1278	226	27	21,144	84.97	97.93	90.99	98.88
		VPF	60	0	2	22,613	100	96.77	98.36	99.99
		CPF	3782	10	76	18,807	99.74	98.03	98.88	99.62
		HPF	4880	1	98	17,696	99.98	98.03	99	99.56
		Average					97.38	97.76	97.5	99.43
		Overall								98.02
Hospital	TabTransformer	Normal	9900	21	852	2435	99.79	92.08	95.78	93.39
		RATSF	1627	14	140	11,427	99.15	92.08	95.48	98.83
		SATSF	0	714	0	12,494	0	0	0	94.59
		SFF	354	30	30	12,794	92.19	92.19	92.19	99.55
		VPF	280	89	25	12,814	75.88	91.8	83.09	99.14
		CPF	0	98	0	13,110	0	0	0	99.26
		HPF	0	81	0	13,127	0	0	0	99.39
		Average					91.75	92.04	91.64	97.73
		Overall								92.07
	TabNet	Normal	9972	23	780	2433	99.77	92.75	96.13	93.92
		RATSF	1639	13	128	11,428	99.21	92.76	95.88	98.93
		SATSF	0	683	0	12,525	0	0	0	94.83
		SFF	356	28	28	12,796	92.71	92.71	92.71	99.58
		VPF	282	78	23	12,825	78.33	92.46	84.81	99.24
		CPF	0	65	0	13,143	0	0	0	99.51
		HPF	0	69	0	13,139	0	0	0	99.48
		Average					92.5	92.67	92.38	97.92
		Overall								92.74
Office	TabTransformer	Normal	21,807	21	2007	11,182	99.9	91.57	95.56	94.21
		RATSF	1282	4	118	33,613	99.69	91.57	95.46	99.65
		SATSF	188	86	18	34,725	68.61	91.26	78.33	99.7
		SFF	8788	87	809	25,333	99.02	91.57	95.15	97.44
		VPF	0	2274	0	32,743	0	0	0	93.51
		CPF	0	245	0	34,772	0	0	0	99.3
		HPF	0	235	0	34,782	0	0	0	99.33
		Average					91.8	91.49	91.12	97.75
		Overall								91.57
	TabNet	Normal	22,054	10	1760	11,193	99.95	92.61	96.14	94.95
		RATSF	1297	2	103	33,615	99.85	92.64	96.11	99.7
		SATSF	190	69	16	34,742	73.36	92.23	81.72	99.76
		SFF	8888	61	709	25,359	99.32	92.61	95.85	97.8
		VPF	0	2017	0	33,000	0	0	0	94.24
		CPF	0	225	0	34,792	0	0	0	99.36
		HPF	0	204	0	34,813	0	0	0	99.42
		Average					93.12	92.52	92.46	98.05
		Overall								92.61

#### Best model selection

A large-scale hyperparameter search was conducted for both TabTransformer and TabNet, generating 1,944 models per target building (auditorium, hospital, office), for a total of 5,832 models. Computational efficiency results (Table 6) indicate that TabTransformer exhibited marginally faster inference, processing an average of 45.25 instances per second (mean inference time: 0.0221 s per instance) compared to TabNet’s 43.48 instances per second (0.0230 s per instance).

**Table 6:** Detection speed by attention-based methods.

Method	Seconds per instances						
	Mean	Std	Min	25%	50%	75%	Max
TabTransformer	0.0221	0.0006	0.0215	0.0218	0.0221	0.0225	0.0228
TabNet	0.023	0.0006	0.0222	0.0226	0.0229	0.0233	0.0236

**Fig. 7:**
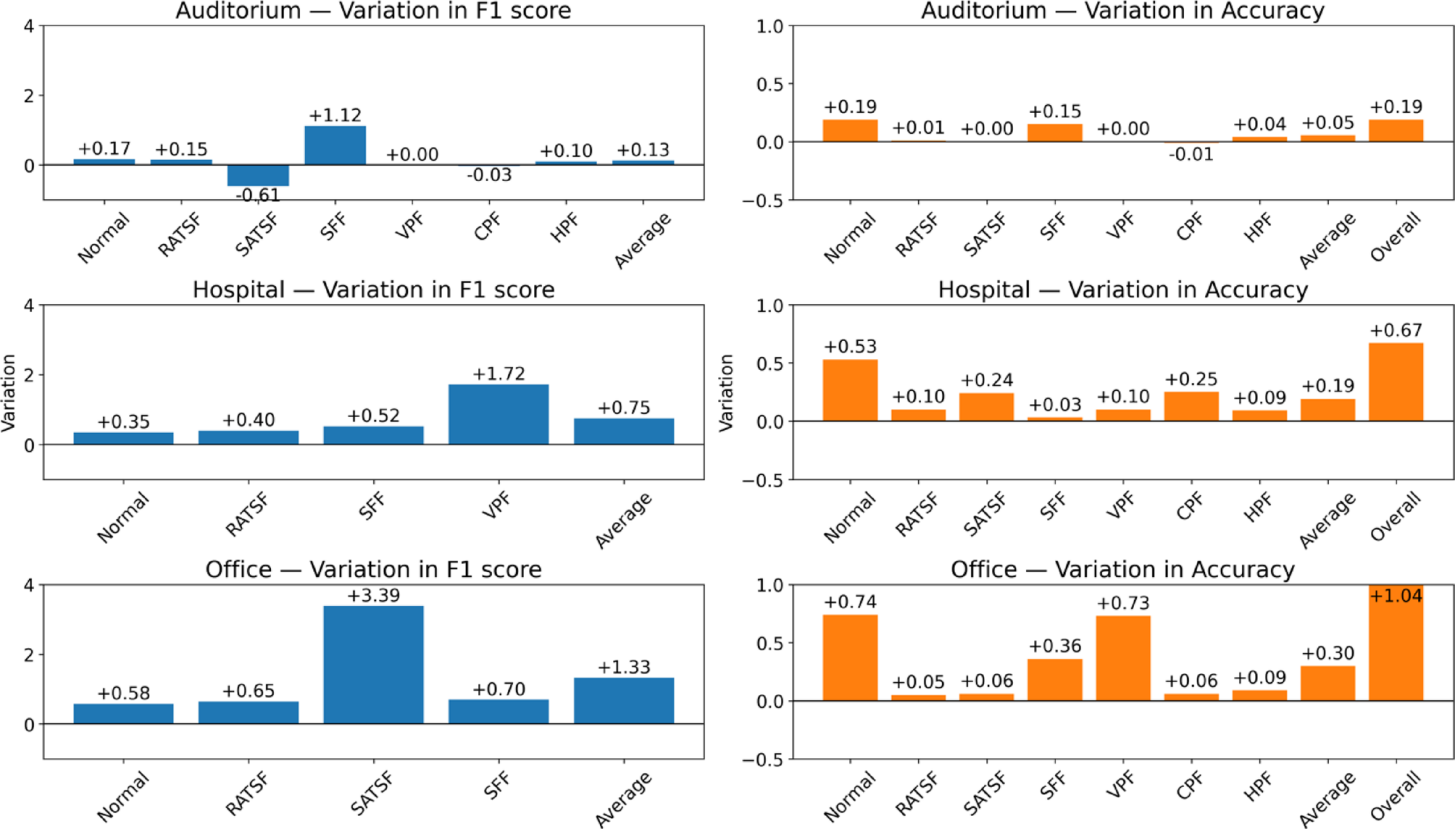
Variation of TabNet and TabTransformer.

Performance variations between the two methods are visualized in Fig. 7, which presents class-wise differences (TabNet − TabTransformer) in F1 score for existing classes and in per-class accuracy for all classes, supplemented by macro averages and overall accuracy. Across all three buildings, TabNet achieved positive mean differences in macro F1 score (+ 0.26% in auditorium, + 0.74% in hospital, + 1.32% in office) and macro per-class accuracy (+ 0.20%, + 0.26%, + 0.30%, respectively). Gains were most notable in classes with moderate to high complexity or imbalanced representation, such as SFF (auditorium: +1.12% F1 score; office: +0.70% F1 score) and SATSF (office: +3.39% F1 score), while improvements for classes with already near-perfect performance were marginal. Accuracy improvements were generally smaller than F1 gains, reflecting that TabNet’s advantage lies more in precision–recall balance for fault categories rather than in the aggregate correct prediction rate.

Although TabTransformer maintained a slight advantage in inference speed, TabNet consistently delivered superior diagnostic performance in terms of both F1 score and accuracy across all buildings and most fault categories. These gains were especially valuable in cases where class imbalance and incomplete fault coverage posed challenges, as seen in the SATSF and VPF classes in the hospital and office datasets. Therefore, considering the trade-off between detection speed and predictive performance, and prioritizing F1 score and accuracy as the primary evaluation metrics, the TabNet-based method was selected as the final model for subsequent analysis and deployment.

### Test results

#### Best model analysis for validation and test sets

**Fig. 8:**
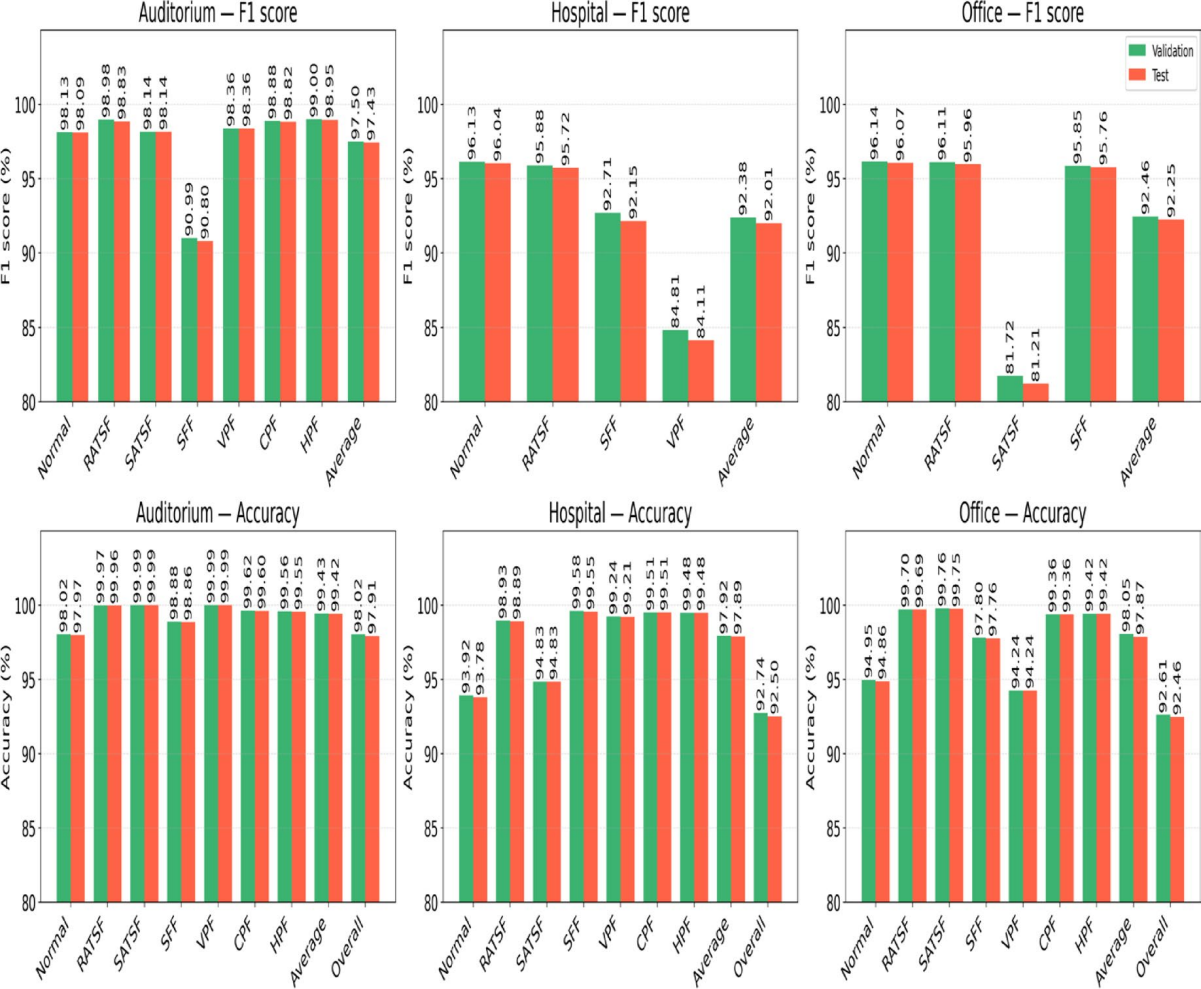
Class-wise F1 score and accuracy comparison of validation and test results.

The best-performing TabNet models selected on the validation set were evaluated on an independent test set to assess generalization and potential overfitting. Figure 8 visualizes the F1 score (top row) and per-class accuracy (bottom row) for all target buildings, and class-wise differences between validation and test performance are summarized in Table 7.

Overall, validation–test differences were small, indicating minimal overfitting. In the auditorium, average F1 decreased by only 0.07% and per-class accuracy by 0.01%, with the largest class-specific drop of − 0.19% F1 for SFF. In the hospital, mean F1 declined by 0.37% and accuracy by 0.03%, with the largest reductions for VPF (− 0.70% F1) and SFF (− 0.56% F1). In the office, average F1 decreased by 0.21% and accuracy by 0.18%, with the greatest single drop in SATSF (− 0.51% F1). Across all buildings, changes in overall accuracy were < 0.25%, reinforcing model stability.

The dataset exhibits building-specific fault coverage: the auditorium includes six faults (RATSF, SATSF, SFF, VPF, CPF, HPF), the hospital three (RATSF, SFF, VPF), and the office three (RATSF, SATSF, SFF). This mismatch in label sets means some faults occur in only one building, producing limited and non-uniform supervision for a unified model across domains. Consequently, performance is lower for SFF in the auditorium, VPF in the hospital, and SATSF in the office.

**Table 7:** Class-wise performance variation.

Class	Auditorium		Hospital		Office	
	F1 score	Accuracy	F1 score	Accuracy	F1 score	Accuracy
Normal	– 0.04	– 0.05	– 0.09	– 0.14	– 0.07	– 0.09
RATSF	– 0.15	– 0.01	– 0.16	– 0.04	– 0.15	– 0.01
SATSF	0	0	–	0	– 0.51	– 0.01
SFF	– 0.19	– 0.02	– 0.56	– 0.03	– 0.09	– 0.04
VPF	0	0	– 0.70	– 0.03	–	0
CPF	– 0.06	– 0.02	–	0	–	0
HPF	– 0.05	– 0.01	–	0	–	0
Average	– 0.07	– 0.01	– 0.37	– 0.03	– 0.21	– 0.18
Overall	–	– 0.11	–	– 0.24	–	– 0.15

#### Attention heat map analysis

##### Feature level temporal analysis

**Fig. 9:**
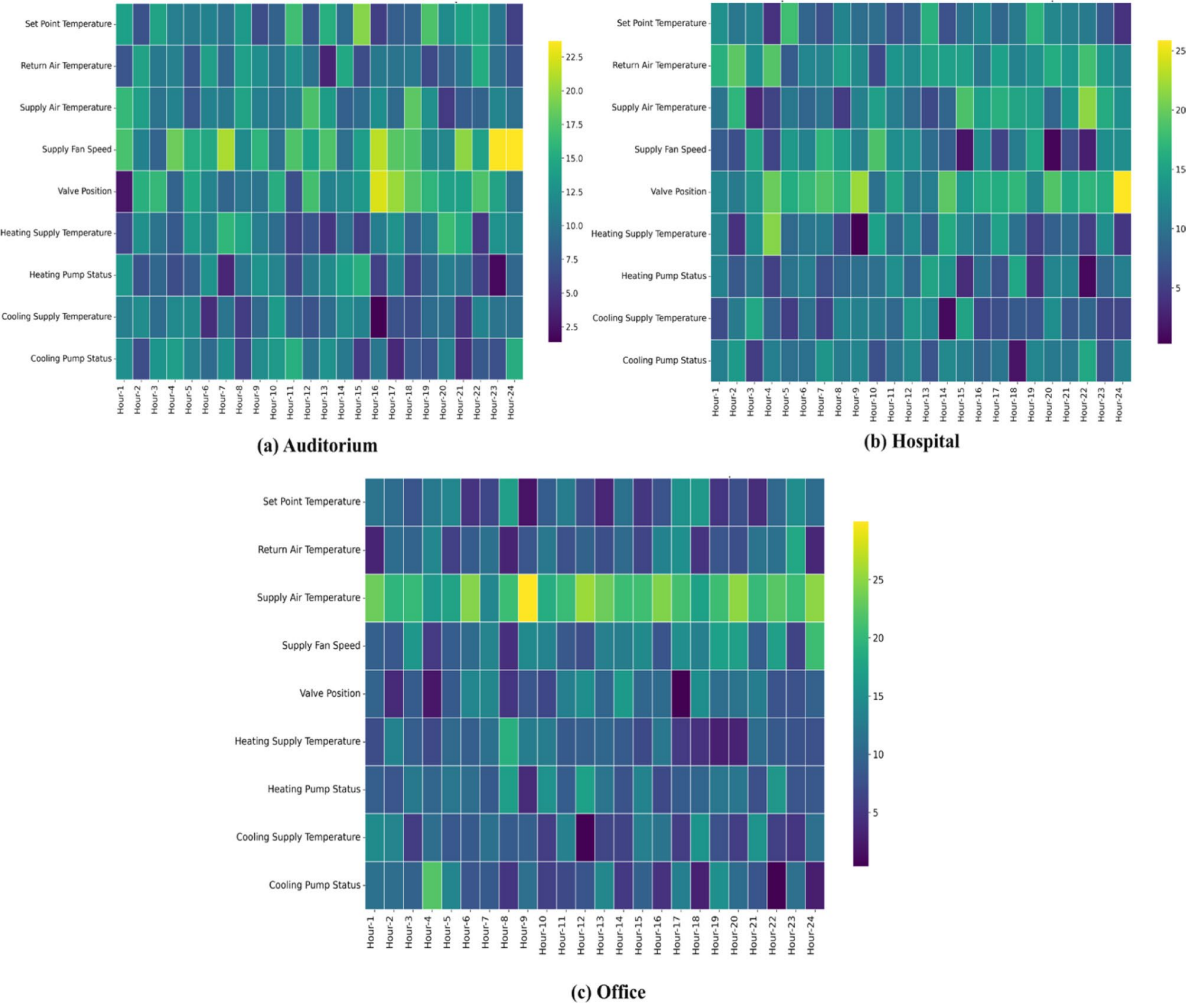
Heat map results of feature temporal analysis in each building data.

Figure 9 presents annotated temporal attention heatmaps—columns normalized to 100% per hour—for (a) auditorium, (b) hospital, and (c) office. Table S.9 provides the corresponding numeric values. Using the same unified TabNet model across buildings, attention redistributes according to each dataset’s characteristics. In the auditorium, which has full coverage of all seven faults, attention is comparatively balanced but increases toward later hours for Valve Position and Supply Fan Speed (means 13.33% and 15.75%; late–early changes of + 4.44 and + 2.69% points), indicating stronger reliance on actuator cues when distinguishing SFF.

In the hospital, where SATSF/CPF/HPF are absent, attention concentrates on Valve Position and Supply Air Temperature (means 15.01% and 13.09%), with the largest late-day increases in Supply Air Temperature (+ 5.58% points) and Valve Position (+ 3.84% points), consistent with the building’s emphasis on VPF. In the office, where VPF/CPF/HPF are absent, Supply Air Temperature dominates (mean 20.64%; +4.76% points), with a secondary rise in Cooling Supply Temperature (+ 3.16% points), aligning with SATSF as the most challenging class. Overall, full fault coverage (auditorium) yields a more balanced temporal profile across sensors, whereas reduced coverage (hospital/office) drives attention to converge on a smaller set of discriminative channels.

##### Feature-class importance analysis

**Fig. 10:**
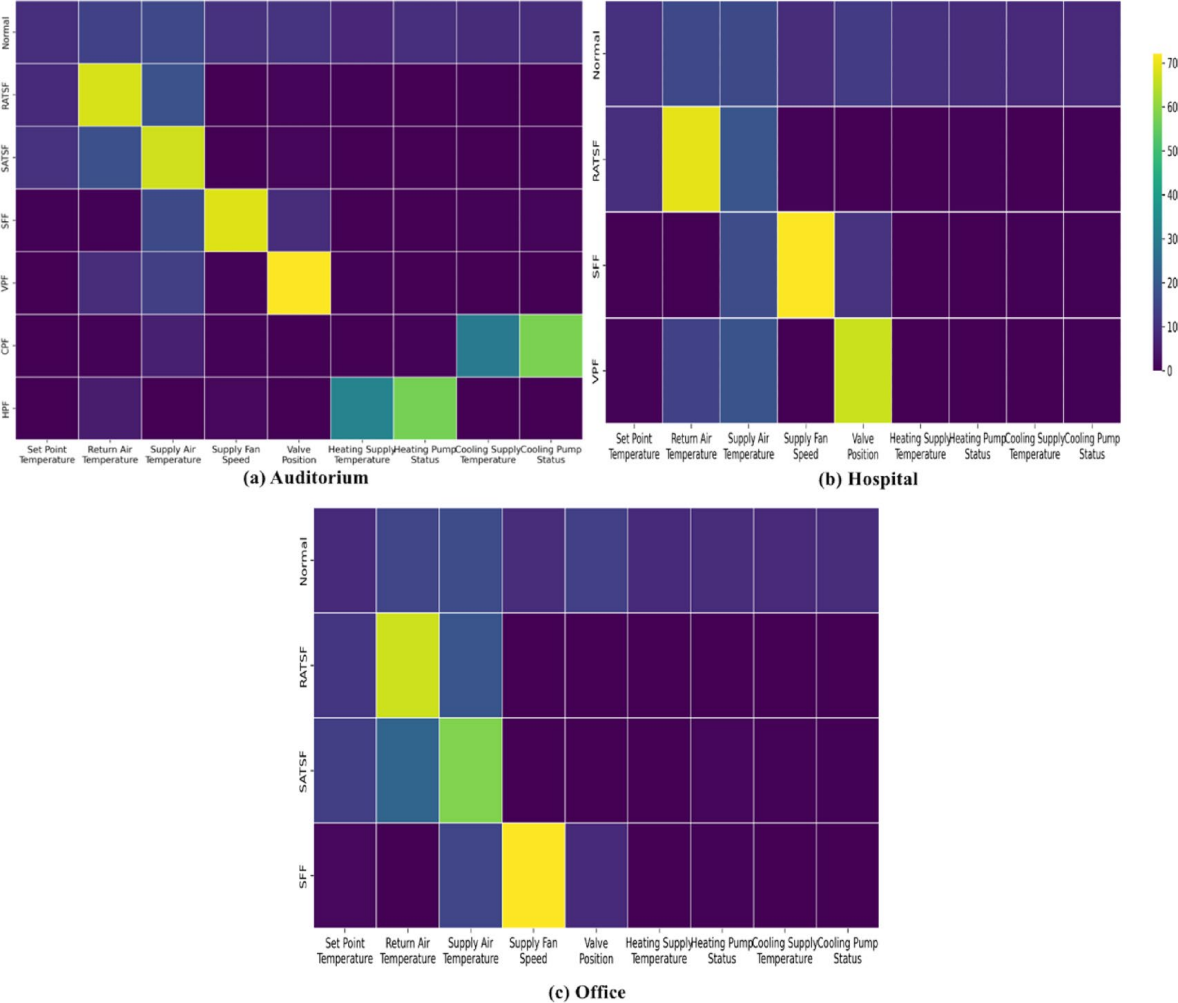
Heat map results of feature-class level analysis in each building.

Class–feature attention heatmaps for (a) auditorium, (b) hospital, and (c) office are presented in Fig. 10 (rows normalized to 100% per class), with the numerical values reported in Table S10. Per-class attention patterns are physically consistent across buildings while reflecting differences in available fault coverage.

In the auditorium, SFF is driven primarily by Supply Fan Speed (68.12%), with Supply Air Temperature (15.17%) and Valve Position (11.58%) as secondary cues (Top1 + Top2 = 83.29%), underscoring the pivotal role of actuator signals. In the hospital, VPF is dominated by Valve Position (64.88%), followed by Supply Air Temperature (18.90%) and Return Air Temperature (13.11%) (Top1 + Top2 = 83.79%), consistent with valve-centric behavior. In the office, SATSF places most weight on Supply Air Temperature (59.85%) and Return Air Temperature (22.03%) (Top1 + Top2 = 81.88%); RATSF emphasizes Return Air Temperature (67.96%) with Supply Air Temperature (18.99%) secondary, and SFF emphasizes Supply Fan Speed (74.39%) with Supply Air Temperature (14.56%).

These heatmaps show attention that is both physics-consistent and coverage-dependent: actuator faults weight actuator channels (SFF→Fan Speed; VPF→Valve Position), and sensor faults weight the corresponding temperatures (SATSF/RATSF→Supply/Return Air). With complete coverage in the auditorium, attention is concentrated (Top1 + Top2 ≈ 80%+) on the decisive channel(s), yielding clearer class separation and higher averages; in the hospital/office, where classes are missing, attention spreads to proxy cues (e.g., temperatures for VPF/SATSF), which explains the lower macro-F1 score.

#### Comparative analysis with other classifiers

##### Selection of comparative methods

To assess the effectiveness of the proposed TabNet-based approach, comparative analyses were performed against three non-attention classifiers: Artificial Neural Networks (ANN), Recurrent Neural Networks with Long Short-Term Memory (RNN-LSTM), and Graph Convolutional Networks (GCN). TabNet was trained on the unified multi-building dataset, whereas the baselines were trained separately for each building. Hyperparameters for all baselines were tuned via grid search. Per building, ANN, GCN, and RNN-LSTM evaluated 162, 216, and 486 configurations, respectively; across the auditorium, hospital, and office datasets this resulted in 2592 trained models.

Table S.7 summarizes, for all methods, the representative fixed settings, the adjustable hyperparameter grids, the number of configurations, and the best configurations selected based on validation-set performance. For completeness, the GCN row also records the fixed graph schema used across all experiments; this schema was specified a priori and was not treated as a hyperparameter. The basic operation of each method’s components—ANN, RNN-LSTM^13^, and GCN^61^—is described in the cited references.

##### Comparative results

Table 8 compares TabNet (transformer-based attention) with conventional baselines (ANN, RNN-LSTM, and GCN) under two regimes: unified multi-building vs. single-building training. On the test set, the unified TabNet improves only when the target building has full fault coverage (auditorium: macro-F1 97.43% vs. 96.82%, + 0.61 pp; accuracy 97.91% vs. 97.37%, + 0.54 pp). With partial coverage, unified training underperforms the single-building TabNet (hospital: macro-F1 92.01% vs. 95.40%; office: 92.25% vs. 96.27%), with the largest drops in VPF (hospital) and SATSF (office).

Relative to single-building non-attention baselines, the single-building TabNet yields the highest macro-F1 in the hospital and office, while overall accuracy is comparable across methods and slightly higher for GCN in those two buildings (hospital 97.81%, office 97.67%). In the auditorium, all single-building methods are tightly clustered (macro-F1 ≈ 96.5–96.9%, accuracy within ≈ 0.2 pp). Overall, unified training is advantageous when fault coverage is complete; with incomplete coverage, single-building training is more reliable.

**Table 8:** Comparison results of best model on test set.

Category	Normal	RTASF	SATSF	SFF	VPF	CPF	HPF	Average	Overall
		TabNet with unified data (Auditorium)							
F1 score	98.09	98.83	98.14	90.80	98.36	98.82	98.95	97.43	–
Accuracy	97.97	99.96	99.99	98.86	99.99	99.60	99.55	99.42	97.91
		TabNet with unified data (Hospital)							
F1 score	96.04	95.72	–	92.15	84.11	–	–	92.01	–
Accuracy	93.78	98.89	94.83	99.55	99.21	99.51	99.48	97.89	92.5
		TabNet with unified data (Office)							
F1 score	96.07	95.96	81.21	95.76	–	–	–	92.25	–
Accuracy	94.86	99.69	99.75	97.76	94.24	99.36	99.42	97.87	92.46
		TabNet with single data (Auditorium)							
F1 score	97.34	98.12	97.96	90.13	97.77	98.13	98.31	96.82	–
Accuracy	96.83	99.53	99.93	98.25	98.31	99.37	98.92	98.73	97.37
		TabNet with single data (Hospital)							
F1 score	97.28	95.90	–	94.40	94.02	–	–	95.40	–
Accuracy	93.96	98.95	94.90	99.58	99.27	99.53	99.49	97.95	97.21
		TabNet with single data (Office)							
F1 score	97.18	96.05	95.95	95.90	–	–	–	96.27	–
Accuracy	94.95	99.72	99.78	97.84	94.28	99.40	99.44	97.92	97.29
		ANN with single data (Auditorium)							
F1 score	97.22	98.03	97.73	89.98	97.74	98.16	98.29	96.74	–
Accuracy	96.77	99.50	99.88	98.21	98.29	99.35	98.89	98.70	97.51
		ANN with single data (Hospital)							
F1 score	95.99	95.63	-	92.02	93.90	–	–	94.39	–
Accuracy	93.71	98.84	94.79	99.51	99.19	99.49	99.46	97.86	97.33
		ANN with single data (Office)							
F1 score	95.98	95.91	91.02	95.62	–	–	–	94.63	–
Accuracy	94.90	99.67	99.71	97.63	94.16	99.33	99.39	97.83	97.16
		RNN-LSTM with single data (Auditorium)							
F1 score	97.31	98.10	97.82	90.09	97.81	98.24	98.36	96.82	–
Accuracy	96.79	99.52	99.90	98.23	98.31	99.37	98.91	98.72	97.52
		RNN-LSTM with single data (Hospital)							
F1 score	96.03	95.66	–	92.07	94.12	–	–	94.47	–
Accuracy	93.75	98.86	94.81	99.52	99.20	99.51	99.47	97.87	97.19
		RNN-LSTM with single data (Office)							
F1 score	96.02	95.95	91.36	95.75	–	–	–	94.77	–
Accuracy	94.94	99.68	99.74	97.76	94.22	99.35	99.41	97.87	97.22
		GCN with single data (Auditorium)							
F1 score	97.06	97.32	97.54	91.73	97.78	97.99	98.77	96.88	–
Accuracy	95.43	99.37	99.54	98.22	98.36	99.41	98.52	98.41	97.53
		GCN with single data (Hospital)							
F1 score	95.62	95.61	–	91.58	93.16	–	–	93.99	–
Accuracy	93.61	98.72	94.75	99.14	99.31	99.12	99.62	97.75	97.81
		GCN with single data (Office)							
F1 score	95.73	95.41	90.76	95.21	–	–	–	94.28	–
Accuracy	95.59	99.68	99.66	98.42	94.28	99.22	99.17	98.00	97.67

## Conclusions

Deep learning models trained on real operating data have shown strong AFDD performance for AHUs, yet much of the literature trains and evaluates on single buildings, limiting transfer to new sites. Many prior approaches also assume a fixed feature schema (same sensors and counts), so accuracy degrades when buildings instrument different variables. To address this, two attention-based tabular models—TabTransformer and TabNet—were trained on a unified multi-building dataset pooling operational data from an auditorium, a hospital, and an office. Hyperparameters were tuned extensively: 3,240 configurations for the attention models (1,296 TabTransformer; 1,944 TabNet), and 2,592 single-building baseline configurations (ANN, RNN-LSTM, and GCN across the three sites). Analyses covered under/overfitting checks, variance across methods, best-model selection, attention heat maps, and classifier comparisons.

The optimized TabNet trained on the unified dataset achieved a macro-F1 of 97.43% and an accuracy of 97.91% for the auditorium, 92.01% and 92.50% for the hospital, and 92.25% and 92.46% for the office. Single-building TabNet baselines reached 96.82% macro-F1 and 97.37% accuracy for the auditorium, 95.40% and 97.21% for the hospital, and 96.27% and 97.29% for the office. These results indicate that unified training is most beneficial when fault coverage is complete (auditorium), whereas single-building training performs better under incomplete coverage (hospital and office). Overall, TabNet ranked highest across settings, with ANN, RNN-LSTM, and GCN close behind. All models showed reliable performance; however, generalization to new buildings depends on fault-class coverage alignment and requires further validation, given potential confounders such as environmental conditions, operating schedules, and differences in sensor configuration and calibration across buildings.

Despite promising results, external validity is limited by the short collection period and the single geographic region. Differences in sensor inventories, fault prevalence, and operating regimes not represented here may yield different outcomes elsewhere. Future studies should broaden temporal and regional coverage to capture seasonal and geographic variation. The unified model offers a clear advantage when the target site’s fault coverage matches the training set, but this advantage narrows under incomplete coverage. Mitigations include coverage-aware training (e.g., presence-gated heads to mask absent classes, class-wise reweighting or post-hoc calibration), targeted augmentation of missing faults, and light per-building fine-tuning; advanced directions include graph attention networks and self-supervised/contrastive pretraining.

## Supplementary Information

Below is the link to the electronic supplementary material.

Supplementary Material 1

## Data Availability

Some or all data, models, or code that support the findings of this study are available from the corresponding author upon reasonable request.
