# Supplementary material for: Fault-class coverage–aligned combined training for AFDD of AHUs across multiple buildings
Source: Sci Rep. 2025 Nov 21;15:41192. doi: 10.1038/s41598-025-24959-9 (PMC12639149; doi:10.1038/s41598-025-24959-9)
Supplement: Supplementary file 1 — Supplementary Material 1 [file 41598_2025_24959_MOESM1_ESM.docx]

**Supplementary Tables**

**Table S.1** Specifications of installed AHUs within the auditorium facility

| Name | Purpose | Cooling coil | | Heating coil | |
| --- | --- | --- | --- | --- | --- |
|  |  | Capacity (W*)* | Flow rate ($\mathrm{LPM}$*)* | Capacity (kcal/h*)* | Flow rate ($\mathrm{LPM}$*)* |
| AHU-01 | B1F ~ 1F Anteroom | 90,500 | 260 | 56,900 | 164 |
| AHU-02 | B1F Orchestra | 65,900 | 189 | 54,200 | 78 |
| AHU-04 | 1F Seating | 226,970 | 651 | 148,400 | 213 |
| AHU-05 | 2F Seating | 83,300 | 239 | 52,200 | 75 |
| AHU-07 | Main stage | 126,000 | 361 | 48,900 | 140 |
| AHU-08 | Left, right stage | 112,300 | 322 | 84,200 | 242 |
| AHU-09 | Rear stage | 57,700 | 166 | 53,500 | 154 |
| AHU-10 | Garnd hall | 228,300 | 654 | 213,400 | 612 |
| AHU-11 | Large theather hall-1 | 140,200 | 402 | 152,200 | 437 |
| AHU-12 | Large theather hall-2 | 140,500 | 403 | 130,100 | 373 |
| AHU-13 | Foyer-1 | 37,900 | 109 | 23,800 | 35 |
| AHU-15 | Multipurpose room | 61,500 | 177 | 49,300 | 142 |
| AHU-16 | Staff cafeteria | 54,200 | 156 | 48,200 | 138 |

**Table S.2** Specifications of installed AHUs within the hospital facility

| Name | Purpose | Cooling coil | | Heating coil | |
| --- | --- | --- | --- | --- | --- |
|  |  | Capacity (W) | Flow rate ($\mathrm{LPM}$) | Capacity (kcal/h) | Flow rate ($\mathrm{LPM}$) |
| AHU-N01 | B1F office zone | 120,306 | 345 | 114,900 | 164 |
| AHU-N02 | B1F food court | 175,838 | 504 | 165,088 | 237 |
| AHU-N03 | B1F common facility | 197,961 | 568 | 194,4453 | 281 |
| AHU-N04 | 1F open lobby | 270,115 | 775 | 279,950 | 803 |
| AHU-N05 | 2F reception area | 131,738 | 378 | 134,429 | 193 |
| AHU-N06 | 3F examination rooms | 358,240 | 961 | 292,516 | 420 |
| AHU-N07 | 4F standard ward | 290,039 | 832 | 245,167 | 352 |
| AHU-N08 | 5F standard war | 319,591 | 916 | 291,779 | 419 |

**Table S.3** Specifications of installed AHUs within the office facility

| Name | Purpose | Cooling coil | | Heating coil | |
| --- | --- | --- | --- | --- | --- |
|  |  | Capacity (W*)* | Flow rate  (LPM*)* | Capacity (kcal/h*)* | Flow rate  (LPM*)* |
| AHU-101 | 1F lobby | 160,393 | 460 | 91,405 | 131 |
| AHU-102 | 1F labarotory | 141,298 | 405 | 98,450 | 142 |
| AHU-103 | 1F office | 244,800 | 702 | 170,107 | 244 |
| AHU-104 | 2F office | 186,616 | 535 | 179,540 | 258 |
| AHU-105 | 3F office/ labarotory | 299,411 | 859 | 276,342 | 397 |
| AHU-107 | 4F labarotory | 214,261 | 615 | 199,285 | 286 |
| AHU-108 | 4F-5F office | 151,480 | 435 | 104,323 | 150 |
| AHU-109 | 4F-5F labarotory | 133,873 | 384 | 129,960 | 187 |
| AHU-110 | 5F labarotory | 158,672 | 455 | 144,932 | 208 |
| AHU-111 | 6F labarotory | 131,870 | 378 | 124,188 | 178 |
| AHU-112 | 6F office | 118,172 | 339 | 84,843 | 122 |
| AHU-201 | B1F meeting room | 214,568 | 615 | 116,130 | 333 |
| AHU-202 | B1F lobby | 262,999 | 754 | 229,104 | 657 |
| AHU-203 | B1F gym | 113,381 | 325 | 58,146 | 167 |
| AHU-204 | B1F restaurant | 139,936 | 402 | 83,214 | 239 |
| AHU-205 | 2F office | 131,401 | 377 | 69,345 | 199 |
| AHU-206 | 3F office | 131,401 | 377 | 69,345 | 199 |
| AHU-207 | 4F office | 133,471 | 383 | 70,551 | 203 |
| AHU-208 | 5F office | 133,471 | 383 | 70,551 | 203 |
| AHU-209 | 6F office | 133,471 | 383 | 70,551 | 203 |

**Table S.4** Labeling basis on seven types of classification

| Category | Description | Numerical ranges |
| --- | --- | --- |
| Normal condition | All parameters listed are within their normal ranges, with no anomalies detected | 1) Return Air Temperature: 16°C – 20°C  2) Supply Air Temperature: 16°C – 22°C  3) Set Point Temperature: 22°C – 23°C  4) Supply Fan Speed: 0 (inactive)  5) Heating Supply Temperature: 20°C – 40°C  6) Cooling Pump Status: 0 (inactive) when cooling is not required  7) Heating Pump Status: 0 (inactive) when heating is not required  8) Valve position varies actively (0–100%) based on heating/cooling needs according to temperature set points |
| Return air temperature sensor fault | Return air temperature sensor produces anomalous readings, such as sudden spikes or drops | Return Air Temperature: outside 16°C – 20°C |
| Supply air temperature sensor fault | Supply air temperature sensor produces faulty readings that deviate from expected trends | Supply Air Temperature: outside 16°C – 22°C |
| Supply fan fault | Inconsistent supply fan speed not matching the system's demand based on set point temperature | Supply Fan Speed: active when unnecessary (should be inactive), or inactive/operating at incorrect speeds when required by set point temperature (22–23°C) |
| Cooling pump fault | Cooling pump operates when no cooling is needed, or fails to operate when cooling is required | 1) Cooling Pump Status:   - 0 (inactive) when cooling is required - 1 (active) when cooling is not needed   2) Supply Air Temperature: <14°C or >25°C |
| Heating pump fault | Heating pump operates when no heating is needed, or fails to operate when heating is required | 1) Heating Pump Status:   - 0 (inactive) when heating is required - 1 (active) when heating is not needed   2) Heating Supply Temperature: <20°C or >40°C  3) Return Air Temperature: <16°C |
| Valve position fault | Valve fails to respond appropriately to control signals | Valve position remains fixed at 0% (fully closed) or 100% (fully open) despite changes in heating/cooling needs indicated by temperature set points |

**Table S.5** Example of annotated sensor data for seven different operational conditions (auditorium scenario)

| Set point temperature | Return temperature | Supply air temperature | Supply fan speed | Valve position | Heating supply temperature | Total heating pump | Heating pump 1 | Heating pump 2 | Heating pump 3 | Cooling supply temperature 1 | Cooling supply temperature 2 | Total cooling pump | Cooling pump 1 | Cooling pump 2 | labeling |
| --- | --- | --- | --- | --- | --- | --- | --- | --- | --- | --- | --- | --- | --- | --- | --- |
| 20 | 21.44 | 17.12 | 100 | 48.08 | - | - | - | - | - | 26.14 | 26.34 | 0 | 0 | 0 | Normal condition |
| 22 | 24.57 | 26.77 | 100 | 100 | - | - | - | - | - | 21.42 | 22.59 | 0 | 0 | 0 | Normal condition |
| 22 | 17.42 | 18.87 | 0 | 0 | 55 | 100 | 0 | 100 | 0 | - | - | - | - | - | Supply fan fault |
| 22 | 17.83 | 18.28 | 0 | 0 | 55.01 | 100 | 0 | 0 | 100 | - | - | - | - | - | Supply fan fault |
| 26 | 21.73 | 21.89 | 0 | 0 | - | - | - | - | - | 18.88 | 19.08 | 0 | 0 | 0 | Cooling pump fault |
| 20 | 23.81 | 20.25 | 50 | 71.43 | - | - | - | - | - | 26.43 | 26.65 | 0 | 0 | 0 | Cooling pump fault |
| 22 | 13.92 | 15.69 | 0 | 0 | 34.5 | 0 | 0 | 0 | 0 | - | - | - | - | - | Heating pump fault |
| 27 | 22.88 | 22.52 | 0 | 0 | 18.9 | 0 | 0 | 0 | 0 | - | - | - | - | - | Heating pump fault |
| 22 | 13.91 | 24.95 | 40 | 48.39 | 44.88 | 110 | 0 | 50 | 60 | - | - | - | - | - | Return air temperature fault |
| 20 | 23.63 | 18.1 | 1 | 100 | - | - | - | - | - | 11.86 | 26.4 | 100 | 100 | 0 | Return air temperature fault |
| 18 | 24.23 | 22.42 | 1 | 42.86 | - | - | - | - | - | 17.01 | 18.89 | 100 | 0 | 100 | Supply air temperature fault |
| 21 | 24.33 | 21.87 | 1 | 44 | - | - | - | - | - | 24.8 | 20.28 | 100 | 0 | 100 | Supply air temperature fault |
| 20 | 24.4 | 25.12 | 40 | 0 | - | - | - | - | - | 25.76 | 19.8 | 60 | 60 | 0 | Valve position fault |
| 28 | 20.27 | 20.91 | 1 | 0 | - | - | - | - | - | 17.59 | 17.02 | 25 | 0 | 25 | Valve position fault |

**Table S.6** Distribution of annotated fault types across building types

| Operational condition | Types of buildings | | | |
| --- | --- | --- | --- | --- |
|  | Auditorium | Hospital | Office | Total |
| Normal condition | 60,219 | 53,757 | 119,402 | 233,378 |
| Return air temperature sensor fault | 1,734 | 8,835 | 6,999 | 17,568 |
| Supply air temperature sensor fault | 404 | 9 | 1,031 | 1,444 |
| Supply fan fault | 6,525 | 1,921 | 47,983 | 56,429 |
| Valve position fault | 311 | 1,525 | 117 | 1,953 |
| Cooling pump fault | 19,292 | 0 | 0 | 19,292 |
| Cooling supply temperature fault | 0 | 1 | 0 | 1 |
| Heating pump fault | 24,890 | 0 | 0 | 24,890 |
| Total | 113,375 | 66,048 | 175,532 | 354,955 |

**Table S.7** Detail of used hyperparameters in each method

| Algorithm | Fixed hyperparameters | Adjustable hyperparameters | Numbers of models | Best hyperparameter |
| --- | --- | --- | --- | --- |
| TabTransformer | AdamW; weight decay 1e-4; GELU; layer norm; residual connections; cosine LR schedule with 5% warm-up | Sequence length: [12, 24, 36, Learning rate: [1e-4, 5e-4, 1e-3, Dropout: [0.1, 0.2, 0.3], Attention heads: [2, 4], Batch size: [32, 64], Epochs: [50, 100, 150], Embedding dimensions: [32, 64, 128, 256] | 1,296 | \|  \| Sequence length: 24, Learning rate: 5e-4, Dropout: 0.3, Attention heads: 2, Batch size: 64, Epochs: 150, Embedding dimensions: 128 \| \| --- \| --- \| |
| TabNet | AdamW; weight decay 1e-4; decision width and attention width set to 64; relaxation parameter 1.5; momentum 0.02; virtual batch size 128; gradient clipping 5.0 | Sequence length: [12, 24, 36, Learning rate: [1e-4, 5e-4, 1e-3, Dropout: [0.1, 0.2, 0.3], Attention heads: [2, 4], Batch size: [32, 64], Epochs: [50, 100, 150], Decision steps: [3, 5, 7], Feature mask sparsity: [0.001, 0.01, 0.1] | 1,944 | Sequence length: 24, Learning rate: 1e-3, Dropout: 0.2, Attention heads: 4, Batch size: 32, Epochs: 150, Decision steps: 3, Feature mask sparsity: 0.01 |
| ANN | ReLU; Softmax output; dropout 0.2 between hidden layers; Xavier initialization; Adam | Hidden layers: [1,2,3]; Nodes per layer: [32,64,128]; Learning rate: [0.0001,0.0005,0.001]; Batch size: [32,64,128]; Epochs: [50,100] | 162 (auditorium) | Hidden layers: 2, Nodes per layer: 64, LR: 0.0005, Batch: 32, Epochs: 100 |
|  |  |  | 162 (hospital) | Hidden layers: 3, Nodes per layer: 32, LR: 0.0005, Batch: 32, Epochs: 100 |
|  |  |  | 162  (office) | Hidden layers: 2, Nodes per layer: 64, LR: 0.001, Batch: 64, Epochs: 100 |
| RNN-LSTM | Unidirectional; return_sequences for all but last layer; Dense(128) penultimate layer; Adam; cross-entropy with class weights | Hidden layers: [1,2,3]; Hidden units: [32,64,128]; Embedding size: [50,100,150]; Dropout: [0.1,0.2,0.3]; Learning rate: [0.0001,0.0005,0.001]; Batch size: [32,64,128]; Epochs: [50,100,150] | 486  (auditorium) | Hidden layers: 2, Hidden units: 64, Embedding size: 150, Dropout: 0.3, LR: 0.0005, Batch: 64, Epochs: 150 |
|  |  |  | 486  (hospital) | Hidden layers: 2, Hidden units: 32, Embedding size: 150, Dropout: 0.3, LR: 0.0001, Batch: 32, Epochs: 150 |
|  |  |  | 486  (office) | Hidden layers: 2, Hidden units: 128, Embedding size: 100, Dropout: 0.2, LR: 0.0001, Batch: 32, Epochs: 150 |
| GCN | Nodes are sensors; edges are the union of physical links and pairs with absolute Pearson correlation > 0.6 (computed on training data), with at most top-3 added correlation edges per node; edge weights are 1.0 for physical links and min–max-normalized correlation for added edges; undirected; self-loops; normalized adjacency; global mean pooling | Layers: [1, 2]; Hidden units: [32, 64, 128]; Dropout [0.1, 0.2, 0.3]; Learning rate [1e-4, 5e-4, 1e-3]; Batch size [32, 64]; Epochs [50, 100] | 216  (auditorium) | Layers: 2, Hidden units: 64, Dropout: 0.2, Learning rate: 5e-4, Batch size: 64, Epochs: 100 |
|  |  |  | 216  (hospital) | Layers: 2, Hidden units: 32, Dropout 0.3, Learning rate: 1e-4, Batch size: 32, Epochs: 100 |
|  |  |  | 216  (office) | Layers: 1, Hidden unit: 128, Dropout: 0.2, Learning rate: 1e-4, Batch size: 32, Epochs: 100 |

**Table S.8** Statistics of F1 score and accuracy in each building

| Metric | Target building | Model | Min | 25% | Median | Mean | 75% | Max | Std |
| --- | --- | --- | --- | --- | --- | --- | --- | --- | --- |
| Average F1 score | Auditorium | TabTransformer | 95.27 | 95.37 | 95.52 | 95.63 | 96.21 | 97.37 | 0.47 |
|  |  | TabNet | 95.42 | 95.68 | 95.84 | 95.89 | 96.92 | 97.5 | 0.52 |
|  | Hospital | TabTransformer | 91.42 | 91.48 | 91.56 | 91.55 | 91.61 | 91.63 | 0.49 |
|  |  | TabNet | 91.98 | 92.12 | 92.22 | 92.24 | 92.33 | 92.38 | 0.44 |
|  | Office | TabTransformer | 90.82 | 90.93 | 91.01 | 91.02 | 91.08 | 91.12 | 0.53 |
|  |  | TabNet | 91.37 | 91.71 | 91.89 | 91.98 | 92.36 | 92.46 | 0.48 |
| Overall accuracy | Auditorium | TabTransformer | 96.33 | 96.62 | 96.81 | 96.89 | 97.42 | 97.83 | 0.39 |
|  |  | TabNet | 96.48 | 96.79 | 96.97 | 97.06 | 97.61 | 98.02 | 0.37 |
|  | Hospital | TabTransformer | 91.35 | 91.68 | 91.86 | 91.9 | 92.02 | 92.07 | 0.45 |
|  |  | TabNet | 91.9 | 92.21 | 92.46 | 92.52 | 92.66 | 92.74 | 0.41 |
|  | Office | TabTransformer | 90.9 | 91.2 | 91.38 | 91.41 | 91.53 | 91.57 | 0.46 |
|  |  | TabNet | 91.45 | 91.85 | 92.06 | 92.16 | 92.35 | 92.61 | 0.42 |

**Table S.9** Numeric values of feature temporal matrix

| Auditorium | Set Point Temperature | Return Air Temperature | Supply Air Temperature | Supply Fan Speed | Valve Position | Heating Supply Temperature | Heating Pump Status | Cooling Supply Temperature | Cooling Pump Status |
| --- | --- | --- | --- | --- | --- | --- | --- | --- | --- |
| Hour-1 | 14.03 | 7.3 | 16.25 | 17.21 | 2.75 | 5.99 | 13.14 | 10.92 | 12.42 |
| Hour-2 | 6.97 | 14.29 | 13.86 | 11.22 | 15.52 | 12.55 | 6.94 | 12.13 | 6.52 |
| Hour-3 | 14.35 | 10.41 | 9.83 | 8.47 | 16.18 | 9.96 | 8.8 | 9.12 | 12.88 |
| Hour-4 | 10.91 | 11.09 | 11.16 | 18.67 | 8.47 | 7.57 | 6.42 | 11.87 | 13.82 |
| Hour-5 | 10.44 | 7.26 | 7.33 | 15.3 | 14.95 | 13.31 | 8.02 | 11.95 | 11.44 |
| Hour-6 | 11.16 | 13.84 | 11.18 | 14.84 | 11.43 | 11.45 | 12.86 | 4.28 | 8.95 |
| Hour-7 | 9.38 | 8.6 | 10.28 | 20.87 | 8.76 | 16.03 | 3.78 | 10 | 12.3 |
| Hour-8 | 13.45 | 13.99 | 14.35 | 12.03 | 10.2 | 14.63 | 10.06 | 5.33 | 5.96 |
| Hour-9 | 6.46 | 12.25 | 10.8 | 15.89 | 9.9 | 10.87 | 12.78 | 8.96 | 12.09 |
| Hour-10 | 8.34 | 9.7 | 9.62 | 9.47 | 15.34 | 9.22 | 11.41 | 13.53 | 13.38 |
| Hour-11 | 16.82 | 12.76 | 11.04 | 17.49 | 6.63 | 5.6 | 6.26 | 7.99 | 15.41 |
| Hour-12 | 7.28 | 9.69 | 17.35 | 14.15 | 16.96 | 7.15 | 10.48 | 7.08 | 9.87 |
| Hour-13 | 15.47 | 3.58 | 13.59 | 17.52 | 11.25 | 4.88 | 11.92 | 9.13 | 12.66 |
| Hour-14 | 9.17 | 14.91 | 8.22 | 9.48 | 11.8 | 9.89 | 14.03 | 12.12 | 10.39 |
| Hour-15 | 19.61 | 6.49 | 9.2 | 13.81 | 13.45 | 5.56 | 15.51 | 11.27 | 5.09 |
| Hour-16 | 5.48 | 10.4 | 12.33 | 21.43 | 22.41 | 10.77 | 5.58 | 1.36 | 10.23 |
| Hour-17 | 8.05 | 9.95 | 9.01 | 17.89 | 20.33 | 12.68 | 11.17 | 6.99 | 3.94 |
| Hour-18 | 8.5 | 10.33 | 18.02 | 17.35 | 17.83 | 8.45 | 5.55 | 6.48 | 7.49 |
| Hour-19 | 17.43 | 6.32 | 12.57 | 11.91 | 15.87 | 10.86 | 8.82 | 9.26 | 6.95 |
| Hour-20 | 12 | 8.33 | 5.19 | 11.71 | 14.25 | 16.62 | 10.84 | 9.7 | 11.35 |
| Hour-21 | 14.16 | 10.12 | 7.77 | 19.88 | 14.13 | 15.01 | 9.98 | 4.74 | 4.21 |
| Hour-22 | 14.95 | 15.22 | 8.36 | 14.2 | 17.56 | 5.02 | 5.78 | 11.32 | 7.58 |
| Hour-23 | 10.09 | 9.45 | 11.47 | 23.53 | 14.34 | 12.71 | 1.82 | 9.91 | 6.7 |
| Hour-24 | 5.6 | 7.09 | 9.47 | 23.67 | 9.5 | 11.06 | 8.81 | 9.49 | 15.31 |
|  |  |  |  |  |  |  |  |  |  |
| Hospital |  |  |  |  |  |  |  |  |  |
| Hour-1 | 12.49 | 13.48 | 3.42 | 15.5 | 15.62 | 6.28 | 9.17 | 12.5 | 11.54 |
| Hour-2 | 10.04 | 11.2 | 10.47 | 12.07 | 20.73 | 0.3 | 10.29 | 12.17 | 12.72 |
| Hour-3 | 10.03 | 4.12 | 13.83 | 19.91 | 7.73 | 15.85 | 10.23 | 11.49 | 6.82 |
| Hour-4 | 6.87 | 12.67 | 10.29 | 13.42 | 14.08 | 9.32 | 13.46 | 9.32 | 10.56 |
| Hour-5 | 8.74 | 11.21 | 8.01 | 13.83 | 8.6 | 13.01 | 9.16 | 14.55 | 12.89 |
| Hour-6 | 17.49 | 14.4 | 4.86 | 9.85 | 9.16 | 8.02 | 16.24 | 12.7 | 7.29 |
| Hour-7 | 6.93 | 13.63 | 7.5 | 8.52 | 17.86 | 17.03 | 14.49 | 0.97 | 13.07 |
| Hour-8 | 10.39 | 13.62 | 18.17 | 1.76 | 12.19 | 12.49 | 3.71 | 16.07 | 11.59 |
| Hour-9 | 12.61 | 9.24 | 14.13 | 13.45 | 14.8 | 10.3 | 10.44 | 6.17 | 8.86 |
| Hour-10 | 9.38 | 13.87 | 15.94 | 5.76 | 14.62 | 15.91 | 7.02 | 6.7 | 10.8 |
| Hour-11 | 11.29 | 10.68 | 14.76 | 11.33 | 15.91 | 10.31 | 16.3 | 7.75 | 1.68 |
| Hour-12 | 17.82 | 11.99 | 13.92 | 16.54 | 10.22 | 5.42 | 3.9 | 7.41 | 12.77 |
| Hour-13 | 12.9 | 15.18 | 7.42 | 0.81 | 17.48 | 8.65 | 12.63 | 13.66 | 11.26 |
| Hour-14 | 11.87 | 13.02 | 13.61 | 6.54 | 14.39 | 9.82 | 12.09 | 7.67 | 11 |
| Hour-15 | 11.57 | 17.07 | 21.19 | 2.63 | 15.13 | 5.27 | 0.86 | 9.44 | 16.83 |
| Hour-16 | 7.81 | 12.96 | 15.52 | 13.49 | 14.93 | 13.58 | 9.14 | 5.86 | 6.72 |
| Hour-17 | 4.15 | 10.82 | 12.27 | 13.46 | 24.9 | 4.55 | 11.78 | 5.95 | 12.12 |
| Hour-18 | 10.37 | 7.78 | 19.04 | 8.29 | 17.26 | 6.26 | 8.08 | 12.81 | 10.11 |
| Hour-19 | 9.71 | 13.85 | 16.47 | 7.4 | 10.6 | 12.24 | 7.44 | 12.4 | 9.89 |
| Hour-20 | 7.41 | 12.79 | 17.16 | 14.32 | 14.77 | 8.32 | 11.19 | 5.18 | 8.86 |
| Hour-21 | 10.76 | 17.91 | 11.98 | 3.96 | 11.6 | 6.01 | 8.88 | 9.6 | 19.3 |
| Hour-22 | 12.58 | 10.14 | 13.92 | 7.3 | 15.84 | 9.37 | 10.88 | 7.54 | 12.43 |
| Hour-23 | 4.37 | 12.67 | 20.23 | 8.75 | 20.4 | 8.33 | 9.9 | 7.85 | 7.49 |
| Hour-24 | 6.03 | 15.53 | 10.16 | 11.1 | 21.33 | 10.05 | 7.76 | 10.36 | 7.68 |
|  |  |  |  |  |  |  |  |  |  |
| Office |  |  |  |  |  |  |  |  |  |
| Hour-1 | 8.07 | 10.62 | 21.76 | 4.84 | 16.25 | 10.16 | 18.89 | 0.55 | 8.87 |
| Hour-2 | 3.88 | 7.99 | 19.59 | 11.33 | 10.72 | 11.17 | 12.9 | 7.12 | 15.3 |
| Hour-3 | 12.24 | 11.17 | 17.51 | 11.25 | 17.56 | 9.34 | 8.49 | 6.76 | 5.68 |
| Hour-4 | 5.43 | 6.96 | 17.31 | 12.96 | 11.03 | 7.5 | 14.7 | 14 | 10.12 |
| Hour-5 | 8.77 | 14.47 | 20.76 | 6.02 | 11.54 | 13.11 | 7.6 | 12.77 | 4.96 |
| Hour-6 | 16.65 | 16.27 | 17.54 | 13.61 | 0.39 | 5.83 | 9.57 | 6.2 | 13.95 |
| Hour-7 | 17.46 | 5.14 | 13.84 | 11.89 | 15.96 | 4.96 | 10.67 | 16.78 | 3.29 |
| Hour-8 | 5.26 | 8.97 | 17.23 | 15.95 | 11.84 | 3.13 | 13.02 | 8.3 | 16.3 |
| Hour-9 | 7.94 | 7.9 | 21.87 | 15.67 | 12.49 | 3.45 | 12.88 | 6.21 | 11.59 |
| Hour-10 | 4.58 | 12.54 | 16.69 | 7.26 | 13.67 | 11.67 | 8.02 | 16.81 | 8.76 |
| Hour-11 | 11.26 | 12.31 | 18.75 | 15.62 | 8.74 | 9.28 | 17.17 | 6.49 | 0.39 |
| Hour-12 | 15.79 | 19.39 | 17.44 | 4.46 | 8.41 | 8.5 | 8.87 | 5.32 | 11.83 |
| Hour-13 | 11.6 | 3.86 | 22.07 | 19.97 | 10.25 | 8.3 | 9.05 | 11.95 | 2.95 |
| Hour-14 | 9.54 | 7.56 | 25.11 | 10.86 | 14.93 | 5 | 11.06 | 10.1 | 5.84 |
| Hour-15 | 11.98 | 16.61 | 22.13 | 7.02 | 11.1 | 7.15 | 9.65 | 10.82 | 3.55 |
| Hour-16 | 4.03 | 13.58 | 22.11 | 4.01 | 14.95 | 12.62 | 8.13 | 5.39 | 15.19 |
| Hour-17 | 8.93 | 15.3 | 18.52 | 11.78 | 7.31 | 10.4 | 9.8 | 11.76 | 6.2 |
| Hour-18 | 7.36 | 7.21 | 22.79 | 17.56 | 7.28 | 7.4 | 10.04 | 8.04 | 12.31 |
| Hour-19 | 1.29 | 8.67 | 25.52 | 6 | 7.96 | 8.22 | 11.92 | 18.73 | 11.69 |
| Hour-20 | 5.67 | 9.43 | 26.51 | 9.87 | 6.14 | 11.81 | 8.8 | 12.16 | 9.6 |
| Hour-21 | 13.93 | 6.37 | 25.72 | 14.69 | 5.59 | 4.07 | 13.86 | 9.35 | 6.43 |
| Hour-22 | 9.66 | 14.58 | 15.08 | 8.62 | 6.34 | 15.92 | 11.19 | 13.13 | 5.47 |
| Hour-23 | 4.74 | 10.13 | 21.3 | 14.58 | 7.94 | 9.55 | 9.13 | 11.26 | 11.36 |
| Hour-24 | 3.42 | 0.98 | 28.14 | 19.16 | 5.74 | 2.48 | 10.09 | 13.31 | 16.68 |

**Table S.10** Numeric values of class feature matrix

| Auditorium | Set Point Temperature | Return Air Temperature | Supply Air Temperature | Supply Fan Speed | Valve Position | Heating Supply Temperature | Heating Pump Status | Cooling Supply Temperature | Cooling Pump Status |
| --- | --- | --- | --- | --- | --- | --- | --- | --- | --- |
| Normal | 9.9 | 16.67 | 15.19 | 8.67 | 12.67 | 9.61 | 9.54 | 8.29 | 9.46 |
| RATSF | 9.54 | 69.8 | 19.23 | 0 | 0 | 0 | 0 | 0.3 | 1.14 |
| SATSF | 9.65 | 18.71 | 71.64 | 0 | 0 | 0 | 0 | 0 | 0 |
| SFF | 0 | 2.03 | 15.17 | 68.12 | 11.58 | 2.77 | 0 | 0 | 0.33 |
| VPF | 1.7 | 9.31 | 14.37 | 0.76 | 73.85 | 0 | 0 | 0 | 0 |
| CPF | 0 | 0.23 | 5.76 | 0.46 | 1 | 0.15 | 0.35 | 32.07 | 59.99 |
| HPF | 0 | 6.4 | 0 | 2 | 1.5 | 31.43 | 57.54 | 0 | 1.14 |
|  |  |  |  |  |  |  |  |  |  |
| Hospital |  |  |  |  |  |  |  |  |  |
| Normal | 10.89 | 14.07 | 15.64 | 7.82 | 11.93 | 11.34 | 10.84 | 9.15 | 8.33 |
| RATSF | 7.62 | 69.65 | 21.73 | 0.44 | 0 | 0.47 | 0 | 0 | 0.1 |
| SFF | 0 | 0.76 | 16.56 | 71.28 | 8.96 | 0.87 | 1.57 | 0 | 0 |
| VPF | 1.26 | 13.11 | 18.9 | 0 | 64.88 | 0.12 | 0.24 | 1.48 | 0 |
|  |  |  |  |  |  |  |  |  |  |
| Office |  |  |  |  |  |  |  |  |  |
| Normal | 9.67 | 14.45 | 13.94 | 9.18 | 12.22 | 8.19 | 11.16 | 10.91 | 10.28 |
| RATSF | 9.18 | 67.96 | 18.99 | 0.42 | 1.19 | 0.61 | 0.71 | 0.61 | 0.33 |
| SATSF | 12 | 22.03 | 59.85 | 0 | 1.62 | 1.66 | 1.31 | 0.25 | 1.3 |
| SFF | 0.01 | 0.2 | 14.56 | 74.39 | 9.06 | 0 | 0 | 0 | 1.77 |
